# FOXA1-dependent PUS1 regulates EIF3b stability in a non-enzymatic pathway mediating prostate cancer bone metastasis

**DOI:** 10.7150/ijbs.100905

**Published:** 2024-08-19

**Authors:** Yongxin Wu, Shengmeng Peng, Bisheng Cheng, Haitao Zhong, Meifeng Cen, Jianhan Fu, Tianlong Luo, Zhenghui Guo, Yiming Lai, Hai Huang

**Affiliations:** 1 Department of Urology, Sun Yat-sen Memorial Hospital, Sun Yat-sen University, Guangzhou 510120, China.; 2 Department of Urology, Nanfang hospital, Southern Medical University, Guangzhou 510515, China.; 3 Guangdong Provincial Key Laboratory of Malignant Tumor Epigenetics and Gene Regulation, Guangdong-Hong Kong Joint Laboratory for RNA Medicine, Urology Department, Sun Yat-Sen Memorial Hospital, Sun Yat-Sen University, Guangzhou 510120, China.; 4 Guangdong Provincial Clinical Research Center for Urological Diseases, Sun Yat-Sen Memorial Hospital, Sun Yat-Sen University, Guangzhou 510120, China.; 5 Department of Urology, The Fifth Affiliated Hospital of Xinjiang Medical University, Xinjiang 830000, China.; 6 Department of Urology, The Sixth Affiliated Hospital of Guangzhou Medical University, Qingyuan People's Hospital, Qingyuan 511518, Guangdong, China.

**Keywords:** Prostate cancer, Bone metastasis, PUS1, EIF3b, TTC3

## Abstract

Bone metastasis is a significant contributor to the poor prognosis in prostate cancer. Recent evidence highlights the pivotal role of pseudouridine synthases in solid tumor progression, yet the specific enzyme driving prostate cancer metastasis remains unidentified. This study uncovers a novel regulatory mechanism of the FOXA1/PUS1/EIF3b signaling axis in prostate cancer bone metastasis. We identified elevated PUS1 expression in prostate cancer tissues, correlating with higher clinical grade and worse prognosis. Knockdown of PUS1 inhibited metastasis independently of its enzymatic activity, with EIF3b acting as a downstream effector, protected from ubiquitin-mediated degradation by PUS1. Overexpression of EIF3b countered the metastasis suppression due to PUS1 knockdown. Additionally, FOXA1 was shown to enhance PUS1 expression by binding to its promoter. Mogroside IV-E, a specific PUS1 inhibitor, demonstrated potent anti-metastatic effects by reducing PUS1 expression. Our findings highlight the FOXA1/PUS1/EIF3b axis as a critical mediator of prostate cancer bone metastasis and suggest that targeting this pathway could be a promising therapeutic strategy.

## Introduction

Prostate cancer is the most common tumor in men^1^, with significant variability in patient prognosis. For men diagnosed with localized prostate cancer, the 10-year survival rate can reach up to 99%. In contrast, patients with distant metastases have a 5-year overall survival rate of only 30%^2^. Bone metastases are the most frequent site of distant metastasis, accounting for over 80% of cases^3^, ^4^. Current treatments for bone metastases can alleviate bone-related events but do not significantly improve overall survival or prevent bone metastasis^5^-^7^. Thus, elucidating the molecular mechanisms of bone metastasis in prostate cancer is crucial for developing targeted therapies.

To date, thirteen pseudouridine synthases have been identified in humans, namely DKC1, PUS1, PUS1L, PUS3, TRUB1, TRUB2, PUS7, PUS7L, PUS10, and RPUSD1-4^8^. Pseudouridine synthases primarily function in RNA post-transcriptional modification, catalyzing the isomerization of uridine to pseudouridine, which is believed to enhance RNA stability and regulate protein translation^9^. Recent studies have highlighted the critical roles of pseudouridine synthases in human diseases and tumorigenesis. For example, PUS7 is highly expressed in glioblastoma, where it promotes tumorigenesis by regulating codon-specific translation of key glioblastoma stem cell factors through tRNA pseudouridylation^10^. In colorectal cancer, DKC1 mediates tumor cell proliferation by stabilizing ribosomal protein mRNAs^11^. Harmful mutations in the gene encoding PUS1, which result in the loss of Ψ at specific tRNA positions, lead to mitochondrial myopathy and sideroblastic anemia (MLASA)^12^. Similarly, mutations in PUS3 and PUS7 cause microcephaly and intellectual disability^13^, ^14^. Notably, pseudouridine synthases can also exert functions independent of their enzymatic activity. For instance, PUS7 promotes colorectal cancer cell metastasis by regulating LASP1^15^. Collectively, these studies underscore the importance of pseudouridine synthases in biological processes and their potential enzymatic and non-enzymatic roles in tumorigenesis.

In addition to PUS1, eukaryotic translation initiation factor 3 (eIF3) is a complex composed of thirteen subunits (eIF3a-3m), forming the largest eIF complex with a molecular weight of approximately 550-700 kDa^16^, ^17^. eIF3 plays crucial roles in protein translation, cell cycle regulation, and tumorigenesis^18^. Increasing evidence suggests that several subunits are aberrantly expressed in cancers and are associated with tumor progression. eIF3h, for instance, facilitates gastric cancer cell growth by suppressing apoptosis^19^. In clear cell renal cell carcinoma (ccRCC), elevated EIF3b expression is linked to a more aggressive tumor phenotype and acts as an independent prognostic indicator^20^. Overexpressed EIF3b in cholangiocarcinoma cells mediates tumor cell survival and migration by inhibiting PCNA ubiquitination^21^. Similar to other tumors^18^, ^22^, ^23^, EIF3b is markedly overexpressed in prostate cancer, contributing to androgen therapy resistance, immune evasion, and metastasis^24^, ^25^. Despite detailed elucidation of EIF3b's oncogenic role in prostate cancer, the regulatory mechanisms governing its expression are still under investigation.

In this study, we present the novel finding that pseudouridine synthase PUS1 is overexpressed in prostate cancer tissues and is positively correlated with adverse patient outcomes for the first time. Mechanistically, PUS1 exerts a non-enzymatic function by directly binding to and stabilizing EIF3b, protecting it from TTC3-mediated ubiquitination and degradation, thereby promoting prostate cancer metastasis. Additionally, the transcription factor FOXA1 can bind to the upstream regions of the PUS1 transcription start site, specifically at 107-118 bp and 1189-1196 bp, thereby promoting the transcription of PUS1. We also identified a small-molecule inhibitor of PUS1, Mogroside IV-E, which dose-dependently inhibits prostate cancer bone metastasis. Overall, our data suggest that the FOXA1/PUS1/EIF3b signaling axis may serve as an effective therapeutic target for treating prostate cancer bone metastasis.

## Materials and methods

### Patients and clinical specimens

Cohort 1 comprised 150 cases of formalin-fixed, paraffin-embedded (FFPE) localized prostate cancer tissues collected from SYSUCC between February 2004 and April 2020. Cohort 2 consisted of 30 cases of benign prostatic hyperplasia tissues, also FFPE, obtained from SYSMH between January 2022 and December 2023. All samples were subjected to immunohistochemical staining and diagnosed by two experienced pathologists. This project received approval from the Institutional Ethical Boards of Sun Yat-sen Memorial Hospital (SYSMH). Clinical patient information was collected in Table S1.

### Cell culture and reagents

Human prostate epithelial cells RWPE-1 and prostate cancer cell lines 22RV1, C4-2, DU145, LNCaP, PC-3, and HEK-293T were acquired from the American Type Culture Collection (ATCC, Manassas, VA, USA). Cells were cultured in PRMI-1640 or DMEM media, each supplemented with 10% fetal bovine serum (FBS; Gibco, Shanghai, China) and antibiotics (100 U/ml penicillin and 100 µg/ml streptomycin; Gibco, Shanghai, China). Mycoplasma contamination was routinely assessed in the cell lines. Purmorphamine, cycloheximide, MG132, and bafilomycin were purchased from Selleck Chemicals, while PUS1 small molecule inhibitor was obtained from MCE.

### Antibodies and plasmids

All antibodies utilized in this study are detailed in Table S2. Plasmids, including Flag-PUS1, HA-EIF3b, HA-ubiquitin, and His-TTC3, were constructed by cloning the corresponding full-length cDNAs into 3xFlag, His, or HA-pcDNA 3.1 vectors. PUS1 or scramble shRNA was introduced into the pLKO.1-puro vector. To create stable cell lines, full-length PUS1-Flag or Luc was incorporated into the pCDH-puro vector.

### Virus packaging and transduction

Virus packaging was performed in HEK-293T cells as described previously^26^. Briefly, lentivirus packaging was achieved by co-transfecting the target plasmids (PUS1-pCDH-puro, shRNA-PUS1 pLKO.1-puro, or control plasmid) with PSPAX2 and PMD2G plasmids. DU145 and PC-3 cells were transduced with the generated viruses supplemented with 10 μg/mL polybrene (IGE) and subjected to antibiotic selection for 2 days. Additionally, PC-3 luciferase stable cells were generated by transduction with Luc-G418 virus followed by G418 (A2513, APExBIO) selection.

### Immunohistochemistry

Immunohistochemical staining of FFPE pathological sections was conducted as reported previously^27^. Briefly, the levels of PUS1 protein were evaluated by staining regions and categorizing them based on the proportion of positive cells (0, 0%; 1, <10%; 2, 10-50%; 3, 51-80%; 4, >80%) and staining intensity (0, negative; 1, weak; 2, moderate; 3, strong). The immunoreactivity score (IRS) was determined by multiplying the percentage score by the intensity score. A high expression was defined as an IRS score greater than 4.

### RNA isolation, real-time quantitative PCR and RNA sequencing

Total RNA was was isolated from the cells using TRIzol (TaKaRa, Dalian, China), and reverse transcription of 1 μg of RNA was carried out using the PrimerScript RT-PCR kit (TaKaRa, Dalian, China). Quantitative PCR was conducted using the LightCycler 480 system with a SYBR Green PCR kit (TaKaRa, Dalian, China), and mRNA levels were calculated using the 2^-ΔΔCt^ method^28^. Primer sequences are provided in Table S3. The clinical characteristics of 13 prostate cancer patients are summarized in Table S4. Library construction and sequencing were performed by RIBOBIO Gene Technology (Guangzhou, China). All RNA sequencing data have been archived in the National Genomics Data Center under accession number HRA002360.

### Flow cytometry

Flow cytometry analysis was performed using the Cytoflex S flow cytometer (Beckman-Coulter). Prostate cancer cells, after transfection with siRNAs, were detached using trypsin and subsequently washed with ice-cold PBS. Following this, the cells were incubated with the Annexin V-FITC apoptosis analysis kit (Tianjin Sungene Biotech Co., Ltd., China) prior to flow cytometric examination. For cell cycle detection, transfected cancer cells were trypsinized, collected gently using PBS, and subsequently fixed in pre-cooled 75% alcohol at -20°C overnight. After centrifugation, discarded the supernatant, and the cells were washed with PBS prior to incubation at 37°C for 30 minutes, following the addition of RNase A. Staining with propidium iodide (PI) was then carried out at 37°C for an additional 30 minutes. Cell apoptosis and cycle were analyzed using Flowjo 10.8.1 software and Modfit 4.05 software.

### Quantification of uridine (U) and pseudouridine (Ψ)

1 μg of RNA was treated with nuclease P1 (N8630, Sigma-Aldrich) in a 20 μl solution of 10 mM ammonium acetate at pH 5.3, and incubated at 42°C for 6 hours. Subsequently, 2 μl of 1 M ammonium bicarbonate and alkaline phosphatase (P4252, Sigma-Aldrich) were added, and the mixture was further incubated at 37°C for an additional 6 hours. The final solution was then diluted to a total volume of 50 μl. The resulting hydrolysates were mixed with three volumes of acetonitrile, centrifuged (15,000 x g, 10 min, 4°C), and the supernatants were subsequently dried and dissolved in 50 µL of a 50% acetonitrile aqueous solution. The nucleosides were separated using a 1290 Infinity II Ultra-Performance LC on an Agilent Poroshell HILIC-z column (2.1x100mm, 2.7µm), and detected using a triple-quadrupole mass spectrometer (Agilent 6470B) in positive ion multiple reaction-monitoring (MRM) mode. The mass transitions of m/z 245.1 to 209.0 and m/z 245.1 to 190.9 for Ψ, and m/z 245.1 to 113.0 and m/z 245.1 to 70.1 for U were monitored and recorded. Quantification of these nucleosides was based on a standard curve generated from the same batch of samples, and the Ψ/U ratios were calculated accordingly.

### RNA interference

siRNAs targeting PUS1, FOXA1, TTC3 and EIF3b, as well as negative controls, were obtained from GenePharma (Suzhou, China) and detailed in Table S5. Transfections were performed using Lipofectamine RNAiMAX (Thermo Fisher Scientific, USA) according to the manufacturer's protocol.

### Cell proliferation assays

Cell viability was assessed using the CCK-8 assay and clonogenic assay, while cell cycle analysis was performed to evaluate cell cycle distribution. Detailed procedures for these experiments are described in previous literature^29^.

### Wound healing assays and transwell migration and invasion assays

Wound healing and transwell assays were conducted to assess the migration and invasion capabilities of prostate cancer cells. Cells that migrated through were photographed and analyzed using a Carl Zeiss Microscopy. Detailed protocols for these experiments are described in previous literature^30^.

### Western blot

Western blotting procedures were carried out as detailed in previous reports^31^. Following incubation with the relevant secondary antibodies (anti-mouse or anti-rabbit, CST, US), protein bands were detected using Immobilon enhanced chemiluminescence (Millipore, USA).

### Immunoprecipitation and Co-IP

The Pierce™ Classic Magnetic IP/Co-IP Kit (Thermo Fisher Scientific, USA) was utilized for immunoprecipitation and co-immunoprecipitation procedures. In brief, the specific antibody was initially combined with the cell lysate to generate an immune complex, which was subsequently captured by magnetic beads. After washing to eliminate unbound material, a low pH elution buffer was employed to dissociate the bound immune complex from the Protein A/G. The resulting immune complex was then subjected to subsequent mass spectrometry or Western blot analysis.

### 3D tumor spheroid invasion assay

DU145 and PC-3 prostate cancer cells were transfected with PUS1-specific shRNA (shPUS1) to generate PUS1 knockdown lines and with scrambled shRNA as control. On the day of the invasion assay, spheroids were carefully transferred to a new 96-well plate pre-coated with 50 µL of Matrigel (Corning, USA) per well. After the Matrigel solidified at 37°C for 30 minutes, 100 µL of complete medium was added to each well. The plates were incubated at 37°C in a 5% CO_2_ atmosphere to allow for spheroid invasion. Spheroids were imaged at 0, 24 hours using an inverted microscope (Olympus, Japan). The invasion area was quantified using ImageJ software (NIH, USA). The invasion index was calculated by measuring the area occupied by invading cells relative to the initial spheroid area.

### Immunofluorescence (IF) staining

Cells were fixed using paraformaldehyde, then blocked with 3% BSA. Primary antibodies were incubated overnight at 4°C. Subsequently, fluorescence-conjugated secondary antibodies (Life Technologies) were applied for 1 hour at room temperature. DAPI (Beyotime, Shanghai, China) staining was performed for 10 minutes, and fluorescence quenching reagent was applied for blocking. Fluorescence images were captured using confocal laser-scanning microscopy at 100× magnification (Leica, Germany).

### Chromatin immunoprecipitation (ChIP)

ChIP experiments were conducted following the instructions provided with the BeyoChIP™ Enzymatic ChIP Assay Kit (P2083S, Beyotime). Briefly, cells were fixed by adding formaldehyde to a final concentration of 1% and incubating at 37°C for 10 minutes. Subsequently, the cells were treated with glycine and incubated at room temperature for 5 minutes. The cells were then washed with PBS containing a protease inhibitor, centrifuged at 1000 × g at 4°C for 2 minutes to collect the cell pellet. The pellet was resuspended sequentially in 1× buffer A and 1× buffer B, and DNA fragmentation was performed using MNase. The MNase reaction was terminated with EDTA, followed by centrifugation and resuspension of the pellet in ChIP Buffer. The nuclear membrane was disrupted by sonication, and the size of the obtained DNA fragments was determined by agarose gel electrophoresis. A 2% input was stored at -20°C, while samples were incubated with 5 μg of primary antibody (FOXA1 or IgG antibody) overnight at 4°C with agitation. The following day, Protein A/G Magnetic Beads/Salmon Sperm DNA were added, and the samples were slowly rotated or agitated at 4°C for 60 minutes to precipitate the protein or corresponding complex recognized by the primary antibody. The bead-antibody-DNA complexes were sequentially washed with buffers of increasing stringency: initially with low salt buffer, followed by high salt buffer, then LiCl wash buffer, and finally TE buffer. Samples were then incubated with freshly prepared Elution Buffer (1% SDS, 0.1M NaHCO_3_) supplemented with NaCl, heated at 65°C for 2 hours to remove the cross-links between proteins and genomic DNA. DNA purification was performed using the Bioteke PCR/DNA Purification Kit (D0033, Beyotime). The extracted DNA was then subjected to real-time PCR analysis using primers designed to assess PUS1 promoter occupancy, as detailed in Table S6.

### DNA gel electrophoresis

PCR products were assessed via agarose gel electrophoresis. In summary, a 1-2% agarose gel was prepared by dissolving agarose in 1× TAE buffer and heating until completely melted. Once cooled to approximately 60°C, ethidium bromide (S3689, Selleck Chemicals) was added to the gel solution to enable visualization of DNA under UV light. The gel was then poured into a casting tray with a well-forming comb and allowed to solidify at room temperature. PCR products were combined with 6x loading dye and then introduced into the wells of the agarose gel. A DNA ladder was included in one of the lanes as a molecular weight marker. The gel was run in 1× TAE buffer at 100-120 volts for 30-45 minutes, or until the dye front had migrated an appropriate distance. Following electrophoresis, the gel was placed on a UV transilluminator and photographed to visualize the bands. The sizes of the PCR products were determined by comparison with the DNA ladder.

### Dual-luciferase reporter assays

The PUS1 promoter region, including the wild-type and mutated sequences, was cloned into the pGL3-Basic vector (Promega). Mutations in the PUS1 promoter were introduced using site-directed mutagenesis (sequence listed in Table S6). DU145 and PC-3 cells were seeded in 24-well plates and co-transfected with 500 ng of the pGL3-PUS1 promoter construct (wild-type or mutant), 50 ng of the pRL-TK plasmid containing Renilla luciferase (Promega) as an internal control, and transfect 200 ng of the FOXA1 expression vector (or the empty vector as a control) using Lipofectamine 3000 (Invitrogen), following the manufacturer's protocol. 48 hours after transfection, cells were lysed, and luciferase activity was assessed using the Dual-Luciferase Reporter Assay System (Promega) according to the manufacturer's instructions. To adjust for variations in transfection efficiency, Firefly luciferase activity was normalized against Renilla luciferase activity. All experiments were conducted in triplicate, and the results are presented as the mean ± SD.

### Bone metastasis xenograft models

All animal experiments were performed in compliance with the ethical standards set forth by the Institutional Ethical Boards of Sun Yat-sen Memorial Hospital (SYSMH), approval number SYSU-IACUC-2023-000989. Four to five-week-old BALB/c nude mice were purchased from Shanghai SLAC Laboratory Animal Co., Ltd. The mouse tail artery injection model for bone metastasis was performed as previously described^30^. Briefly, for PUS1 knockdown *in vivo* experiments, each mouse was injected with 2×10^6 PUS1 knockdown or negative control cells via the tail artery, with 10 mice per group. Tumor growth and bone metastasis were monitored weekly using a bioluminescent imaging system (Bruker MI). For drug treatment *in vivo* experiments, mice were injected with 2×10^6 PC-3 luciferase-expressing tumor cells via the tail artery. When fluorescence signals were detected in the third week, Mice were allocated at random into three groups, with four mice in each group. The control group received intraperitoneal injection of PBS, while the treatment groups received Mogroside IV-E (10 mg/kg and 20 mg/kg) in PBS. Weekly monitoring of bone metastasis was conducted using a bioluminescent imaging system (Bruker MI). X-ray images were captured at an exposure of 10 sec and 35 keV. Finally, harvested tissues were preserved in formalin and decalcified.

### Data analysis

All statistical analyses were performed using SPSS version 26.0 or GraphPad Prism 8.0 software. The two-tailed Student's t-test was employed to assess differences between two groups, whereas one-way ANOVA with Tukey's post hoc test was employed for comparisons involving more than two groups. The correlation between PUS1 expression and clinicopathological characteristics in our cohort as well as in TCGA PRAD data was assessed using Pearson's correlation test. Overall survival (OS) and progression-free survival (PFS) curves were constructed using Kaplan-Meier methods and assessed with the log-rank test. Hazard ratios (HR) and 95% confidence intervals (CIs) for survival were calculated using a Cox proportional hazards model.

## Results

### PUS1 exhibits a pronounced upregulation in PRAD, correlating significantly with adverse prognosis

**Figure 1:**
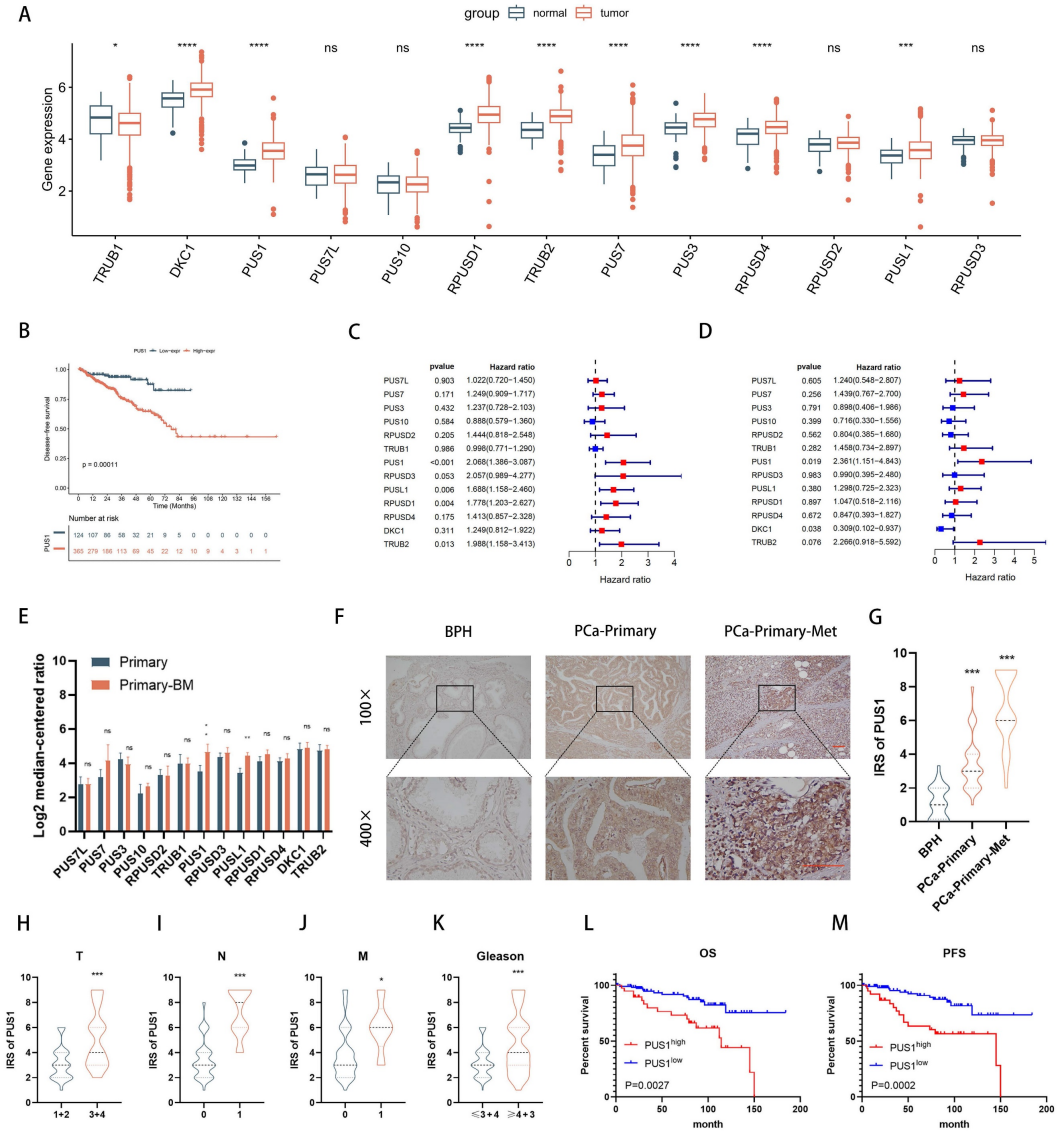
PUS1 exhibits a pronounced upregulation in PRAD, correlating significantly with adverse prognosis. **A** The expression levels of the 13 pseudouridine synthases in TCGA-PRAD. **B** DFS of patients with different PUS1 expression levels. **C&D** Univariate and multivariate Cox regression analysis of the risk assessment for the independent association of 13 enzymes with the prognosis of prostate cancer patients. **E** Expression differences of the 13 enzymes in primary prostate cancer and primary prostate cancer with bone metastasis. **F&G** Representative immunohistochemical staining images of PUS1 and corresponding IRS score statistics in BPH, primary prostate cancer without metastasis, and primary prostate cancer with metastasis. Scale bar, 20 μm. **H-K** Differential expression of PUS1 IRS in cohort 2 correlates with tumor Gleason score, TNM staging, and histological grade. **L&M** Kaplan-Meier curves showing PFS (M) and OS (L) in prostate cancer patients with different PUS1 expression levels in cohort 2. ns: no significance. *p<0.05, **p<0.01, ***p<0.001, ****p<0.0001.

To delineate the roles of pseudouridine-modifying enzymes in prostate cancer, we examined their expression patterns within the TCGA-PRAD database, encompassing a cohort of 13 identified enzymes. Our analysis unveiled a notable upregulation of DKC1, PUS1, RPUSD1, TRUB2, PUS7, PUS3, RPUSD4, and PUSL1 in prostate cancer tissues (Fig. 1A). Strikingly, patients exhibiting elevated levels of PUS1, PUS3, PUSL1, RPUSD1-4, and TRUB2 demonstrated a significantly shorter disease-free survival, as evidenced by combined analysis with tumor disease-free survival data (Fig. 1B and Fig. S1A-L). Furthermore, leveraging both univariate and multivariate logistic regression analyses (Table S7), we identified PUS1 as an independent prognostic indicator in prostate cancer patients (Fig. 1C&D). Transcriptomic scrutiny of PUS1 expression across six prostate cancer *in situ* foci, including six foci with bone metastases, unveiled heightened PUS1 levels in the latter (Fig. 1E, Table S3). Subsequent immunohistochemical staining of pathological sections from 150 cases underscored elevated PUS1 expression in tumors relative to benign prostatic hyperplasia (BPH) (Fig. 1F&G). Intriguingly, within tumor specimens, PUS1 expression positively correlated with tumor grade, stage, and poor prognosis (Fig. 1H-M), providing compelling insights into its clinical relevance and prognostic potential in prostate cancer.

### Elevated expression of PUS1 in prostate cancer cells leads to significant inhibition of tumor cell invasion and migration upon PUS1 knockdown *in vitro* and *in vivo*

**Figure 2:**
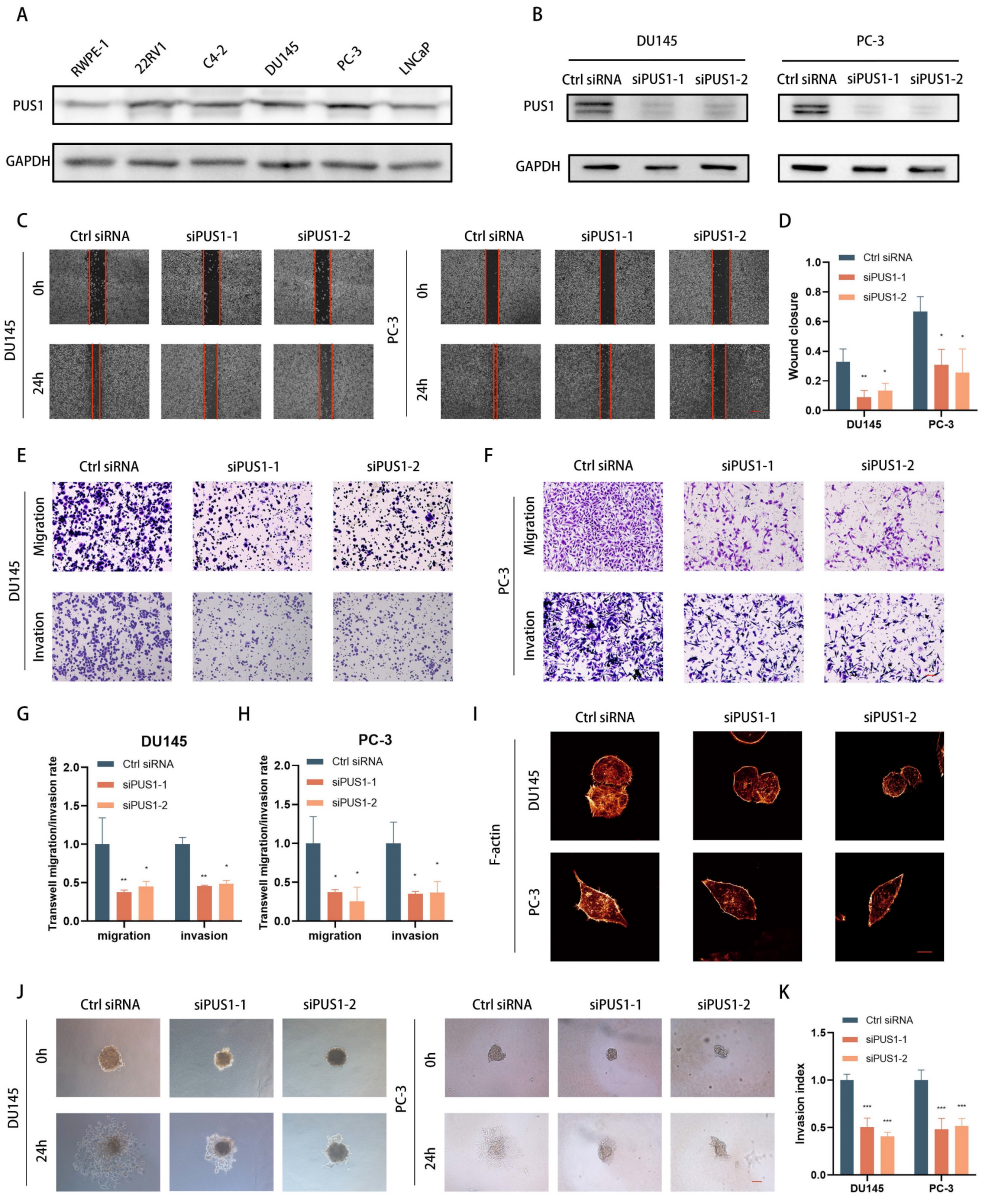
Knockdown of PUS1 Inhibits Invasion and Migration of Prostate Cancer Cells *In Vitro*. **A** Western blot analysis of PUS1 expression in the prostate epithelial cell line (RWPE-1) and prostate cancer cell lines, with GAPDH used as the loading control. **B** Western blot analysis showing PUS1 protein levels in DU145 and PC-3 cells following the treatments indicated. **C&D** Representative images (C) and bar graph analysis (D) of wound healing assays in DU145 and PC-3 cells following the indicated treatments. Scale bar, 20 μm. **E-H** Representative images (E&F) and bar graph analysis (G&H) of transwell migration/invasion assays in DU145 and PC-3 cells following the indicated treatments. **I** Representative immunofluorescence images of F-actin in DU145 and PC-3 cells following the indicated treatments. Scale bar, 5 μm. **J&K** Representative images (J) and bar graph analysis (K) of 3D tumor spheroid invasion assays in DU145 and PC-3 cells following the treatments indicated. Scale bar, 20 μm. All data are presented as the mean ± standard deviation (SD). *p<0.05, **p<0.01, ***p<0.001.

In various prostate cancer cell lines (such as 22RV1, C4-2, DU145, LNCaP, and PC-3), as well as benign prostate hyperplasia cell line RWPE-1, we examined PUS1 expression using qPCR and Western blot. We observed a significant increase in PUS1 expression in tumor cell lines (Fig. 2A). Knocking down PUS1 resulted in a noticeable decrease in the invasion and migration capabilities of tumor cells, as demonstrated by scratch assays and transwell assays (Fig. 2B-H), while proliferation, cell cycle, and apoptosis of tumor cells remained unaffected (Fig. S2A-F). Furthermore, through immunofluorescence staining of F-actin, we observed a decrease in fluorescence intensity following the knockdown of PUS1 (Fig. 2I). Additionally, using a 3D tumor spheroid Matrigel invasion assay, we found that the invasive capacity of the cells significantly decreased with the reduced expression of PUS1 (Fig. 2J&K).

**Figure 3:**
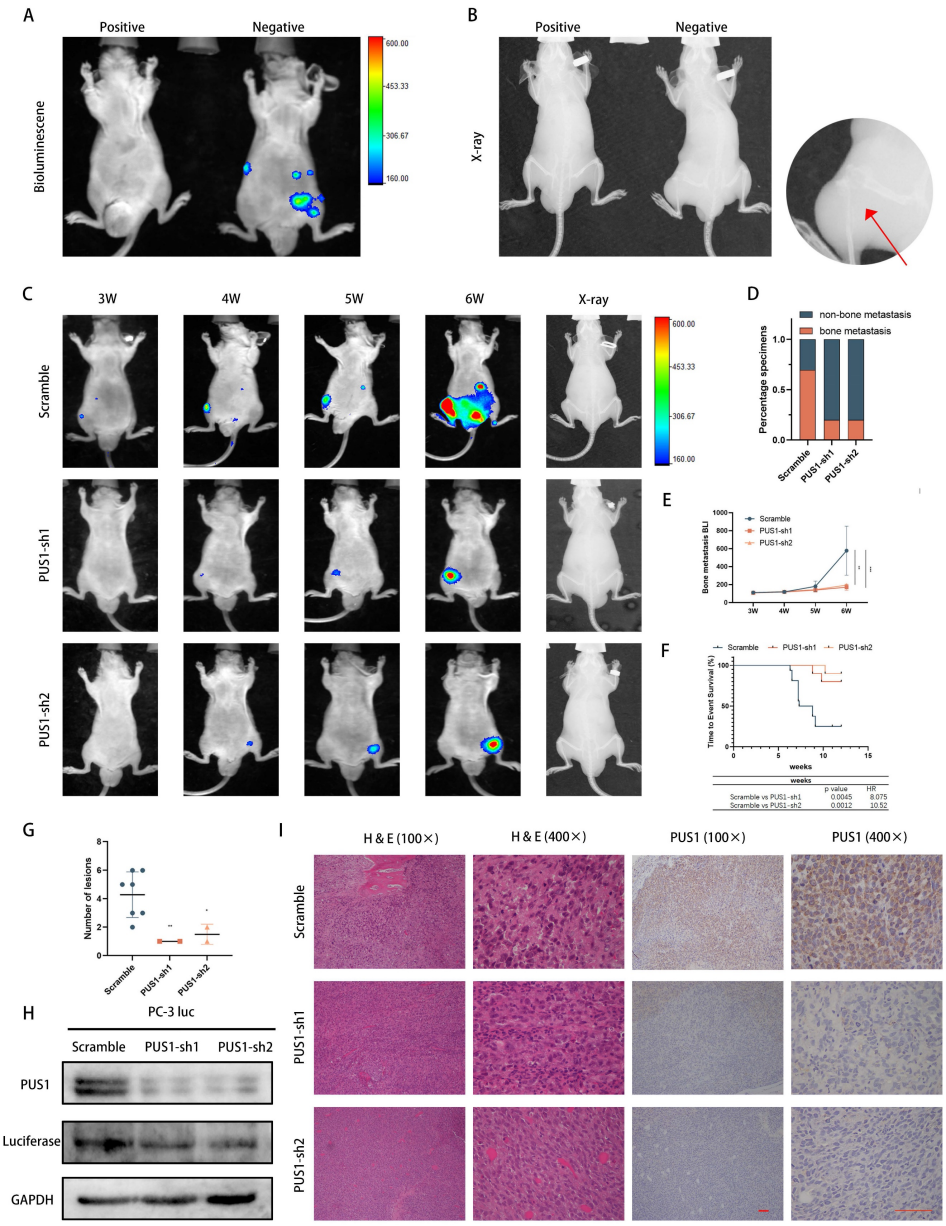
Knockdown of PUS1 Inhibits Bone Metastasis of Prostate Cancer *In Vivo*. **A&B** Bioluminescence imaging and X-ray of bone metastasis following tumor cell tail artery injection, with bone metastases indicated by red arrows. **C** PC-3 cells with PUS1 knockdown and control groups carrying luciferase were injected into the tail artery of nude mice. Bone metastasis was monitored weekly using the IVIS system, and representative bioluminescence imaging results for each group of nude mice are shown. **D** Bar graph showing bone metastasis rates in different treatment groups. **E** Bioluminescence quantification of bone metastatic lesions in different groups of nude mice. **F** Survival Kaplan-Meier curves for each group of mice. **G** The number of metastases in each indicated group (n=10/group). **H** Western blot analysis of PUS1 and luciferase protein levels in tumor tissues from bone metastatic lesions in nude mice, with GAPDH used as a loading control. **I** Immunohistochemical staining of PUS1 and luciferase as labeled, along with representative HE staining of lesions in the indicated groups. Scale bar, 20 μm. *p<0.05, **p<0.01, ***p<0.001.

To further validate the *in vivo* function of PUS1, we initially engineered PC-3 cell lines expressing fluorescent enzymes. Subsequently, using lentivirus-mediated stable knockdown of PUS1, we confirmed knockdown efficiency and then established a mouse model of bone metastasis by intravenous injection of tumor cells via the tail artery (Fig. 3A&B). Real-time monitoring of tumor metastasis in mice was conducted weekly through fluorescence imaging. Consistent with our *in vitro* experiments, significant reduction in the probability of bone metastasis in mice was observed after PUS1 knockdown, with markedly weaker fluorescence intensity in metastatic foci compared to the negative control group. Micro-CT imaging revealed a significant decrease in the size of osteolytic metastatic lesions upon PUS1 knockdown, accompanied by a marked improvement in overall prognosis in the mice. (Fig. 3C-G). Furthermore, we dissected bone metastatic lesions and validated PUS1 expression through H&E staining and IHC staining. Clearly, PUS1 expression was markedly higher in the control group (Fig. 3H&I). Collectively, these findings indicate that PUS1 promotes tumor cell bone metastasis both *in vivo* and *in vitro*.

### PUS1 promotes tumor cell migration and invasion through a non-pseudouridine synthase modification-dependent mechanism

**Figure 4:**
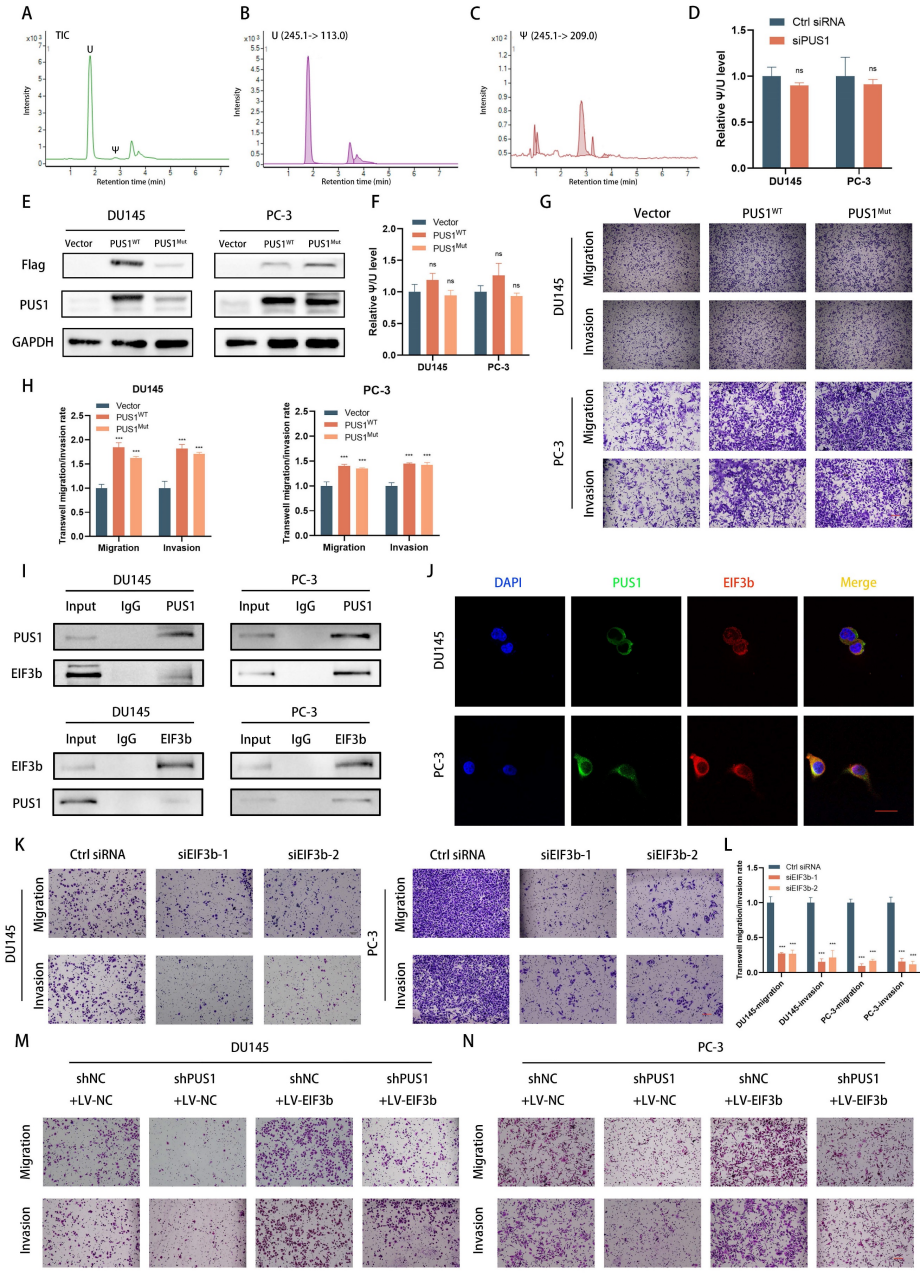
PUS1 promotes tumor cell migration and invasion through a non-pseudouridine synthase modification-dependent mechanism. **A-C** Representative LC/MS/MS chromatograms depicting Total Ion Current (TIC), uridine, and pseudouridine modifications. **D** Quantification of Ψ modification after PUS1 knockdown in DU145 and PC-3 cells. **E&F** Western blot results showing the overexpression of wild-type PUS1, mutant PUS1, and control in DU145 and PC-3 cells, along with quantification of Ψ modification in different groups. **G&H** Representative transwell invasion and migration assay images of DU145 and PC-3 cells treated as indicated, accompanied by corresponding bar graphs for statistical analysis. Scale bar, 20 μm. **I** Immunoprecipitation analysis showing the interaction between endogenous PUS1 and EIF3b in DU145 (left) and PC-3 (right) cells. **J** Representative confocal immunofluorescence images showing colocalization of PUS1 (green) and EIF3b (red) in DU145 and PC-3 cells. Scale bar, 5 μm. **K-N** Representative transwell invasion and migration assay images of DU145 and PC-3 cells treated as indicated, accompanied by corresponding bar graphs for statistical analysis. Scale bar, 20 μm. ns: no significance, ***p<0.001.

Given that PUS1 is one of the 13 pseudouridine modification enzymes, its regulation of tumor cell invasion and migration through its enzymatic reaction remains unknown. Previous literature has reported that pseudouridine synthase PUS7, through a non-enzymatic pathway, interacts with LASP1, stabilizing the latter and promoting colorectal cancer cell metastasis. Therefore, we knocked down PUS1 expression in DU145 and PC-3 cell lines and collected RNA from tumor cells treated differently. After degrading RNA into individual nucleosides and detecting pseudouridine modification levels through mass spectrometry, we found that knocking down PUS1 expression significantly had no apparent effect on overall RNA pseudouridylation levels (Fig. 4A-D).

Furthermore, based on previous literature reporting on PUS1's enzymatic sites^32^, we constructed wild-type PUS1 plasmids and PUS1 mutant plasmids (D146A, Y201F, I294L, R295K, and L333I). These constructs were then transfected into DU145 and PC-3 cell lines. We observed that overexpression of wild-type PUS1 slightly increased tumor cell RNA pseudouridylation levels, while mutants showed no significant changes (Fig. 4E&F). However, both wild-type and mutant forms significantly enhanced tumor cell invasion and migration capabilities, suggesting that the enzymatic function of PUS1 has no apparent effect on tumor cell invasion and migration (Fig. 4G&H).

Since PUS1 does not primarily exert its function through enzymatic pathways, we hypothesize that PUS1 may function through interactions with other molecules. Using co-immunoprecipitation experiments to detect PUS1-interacting molecules, mass spectrometry results indicated that the interacting proteins include EIF3b and KRT1 (Fig. S3A). Given the important role of EIF3b in prostate cancer (Fig. S3B-D) 24, 25, we focused on the contribution of EIF3b to PUS1-mediated prostate cancer metastasis. Subsequently, we conducted co-immunoprecipitation experiments and found that PUS1 could pull down EIF3b in DU145 and PC-3 cell lines, and vice versa. Further immunofluorescence staining revealed co-localization of PUS1 and EIF3b (Fig. 4I&J).

To further clarify that EIF3b is downstream of PUS1 regulation, we knocked down EIF3b in DU145 and PC-3 cell lines and observed a significant decrease in the migration and invasion abilities of the tumor cells. Furthermore, we found that the impaired migration and invasion abilities of tumor cells due to PUS1 knockdown could be rescued by overexpressing EIF3b (Fig. 4K-N and Fig. S3E&F). These results indicate that PUS1 promotes prostate cancer metastasis primarily through its interaction with EIF3b in a non-enzymatic manner.

### PUS1 enhances the stability of EIF3b protein

**Figure 5:**
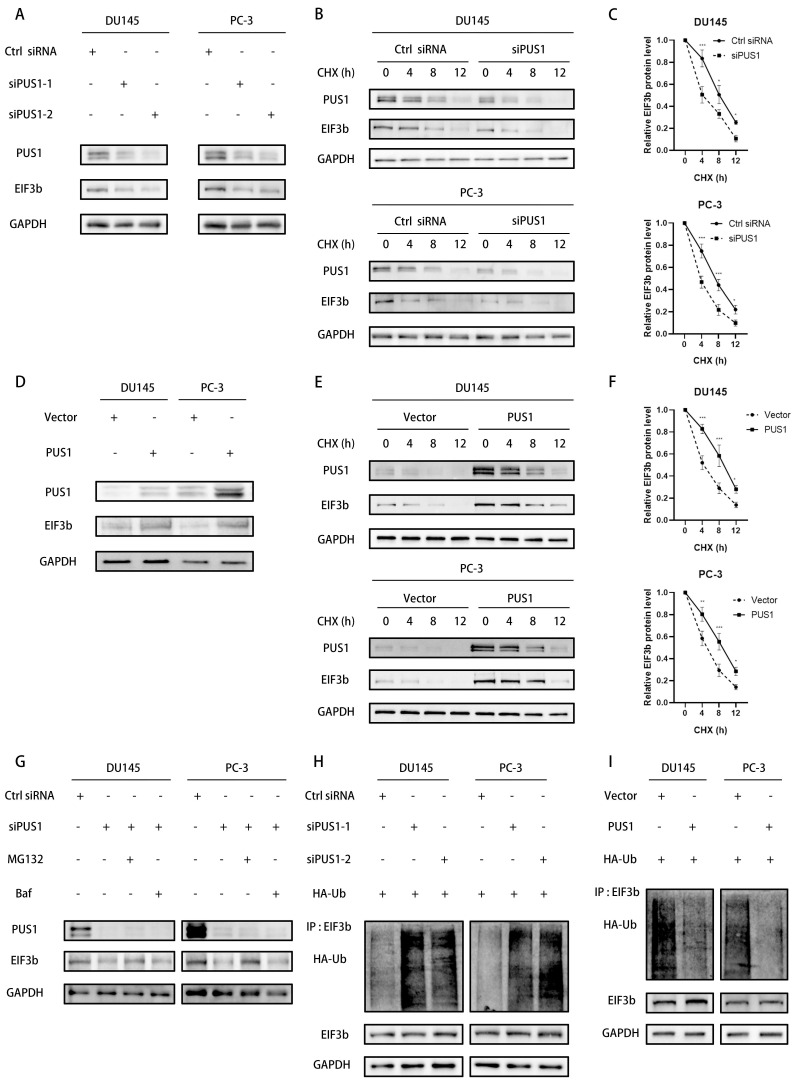
PUS1 enhances the stability of EIF3b protein. **A** Western blot analysis of PUS1 and EIF3b protein expression after transfection with PUS1-siRNA. **B&C** Western blot analysis of EIF3b protein levels at different time points in DU145 and PC-3 cells transfected with si-Ctrl or si-PUS1 after treatment with cycloheximide (CHX, 20 μg/mL), along with corresponding quantification of protein expression. **D** Western blot analysis of PUS1 and EIF3b protein expression after overexpressing PUS1. **E&F** Western blot analysis of EIF3b protein levels at different time points after overexpression of PUS1 in DU145 and PC-3 cells treated with cycloheximide (CHX, 20 μg/mL), along with quantification of EIF3b protein expression. **G** Western blot analysis of DU145 and PC-3 cells transfected with PUS1-siRNA or control, subsequent to treatment with DMSO, MG132 (10 μg/mL), or Baf for a duration of 4 hours. **H&I** Immunoprecipitation analysis of ubiquitination levels of EIF3b in DU145 and PC-3 cells after knockdown (H) or overexpression (I) of PUS1, and co-transfection with HA-Ub and EIF3b plasmids. * p<0.05, ** p<0.01, ***p<0.001.

The experiments above initially found that PUS1 can interact with EIF3b. We further examined whether PUS1 affects the protein levels of EIF3b. Western blot results showed that knocking down PUS1 in prostate cancer cells reduced the protein levels of EIF3b, while overexpression of PUS1 significantly upregulated EIF3b expression (Fig. 5A&D). However, EIF3b mRNA levels were not significantly altered following PUS1 knockdown or overexpression (Fig. S4A-D). This suggests that PUS1 may influence EIF3b protein levels through post-transcriptional modification after transcription.

To further confirm that PUS1 increases EIF3b protein stability, we treated tumor cells with chlorhexidine (CHX), a protein synthesis inhibitor, while knocking down or overexpressing PUS1. We collected cell lysates and detected protein degradation levels through Western blot. Knocking down PUS1 reduced EIF3b expression levels and shortened its half-life, while overexpressing PUS1 significantly prolonged EIF3b half-life (Fig. 5B&C, E&F). This indicates that PUS1 acts as a positive regulator of EIF3b protein stability.

In eukaryotic cells, the degradation of proteins commonly occurs through two pathways: the ubiquitin-proteasome system and the autophagy process. To elucidate the degradation pathway of EIF3b, we employed inhibitors targeting these pathways, namely the ubiquitin-proteasome pathway inhibitor MG132 and the autophagy inhibitor bafilomycin (Baf), in PUS1-knockdown prostate cancer cells. We found that MG132, rather than Baf, inhibited the degradation of EIF3b upon PUS1 knockdown (Fig. 5G). To further determine whether PUS1 stabilizes EIF3b by promoting its de-ubiquitination, we co-transfected EIF3b and HA-ubiquitin plasmids into both PUS1-knockdown and overexpressing cells. Pull-down assays followed by western blot analysis revealed significant ubiquitination of EIF3b in the PUS1-knockdown group, whereas in PUS1-overexpressing tumor cells, EIF3b exhibited markedly reduced polyubiquitination (Fig. 5H&I). These results suggest that PUS1 protects EIF3b from ubiquitin-dependent proteasomal degradation.

### PUS1 protects EIF3b from TTC3-mediated ubiquitination degradation through competitive binding

**Figure 6:**
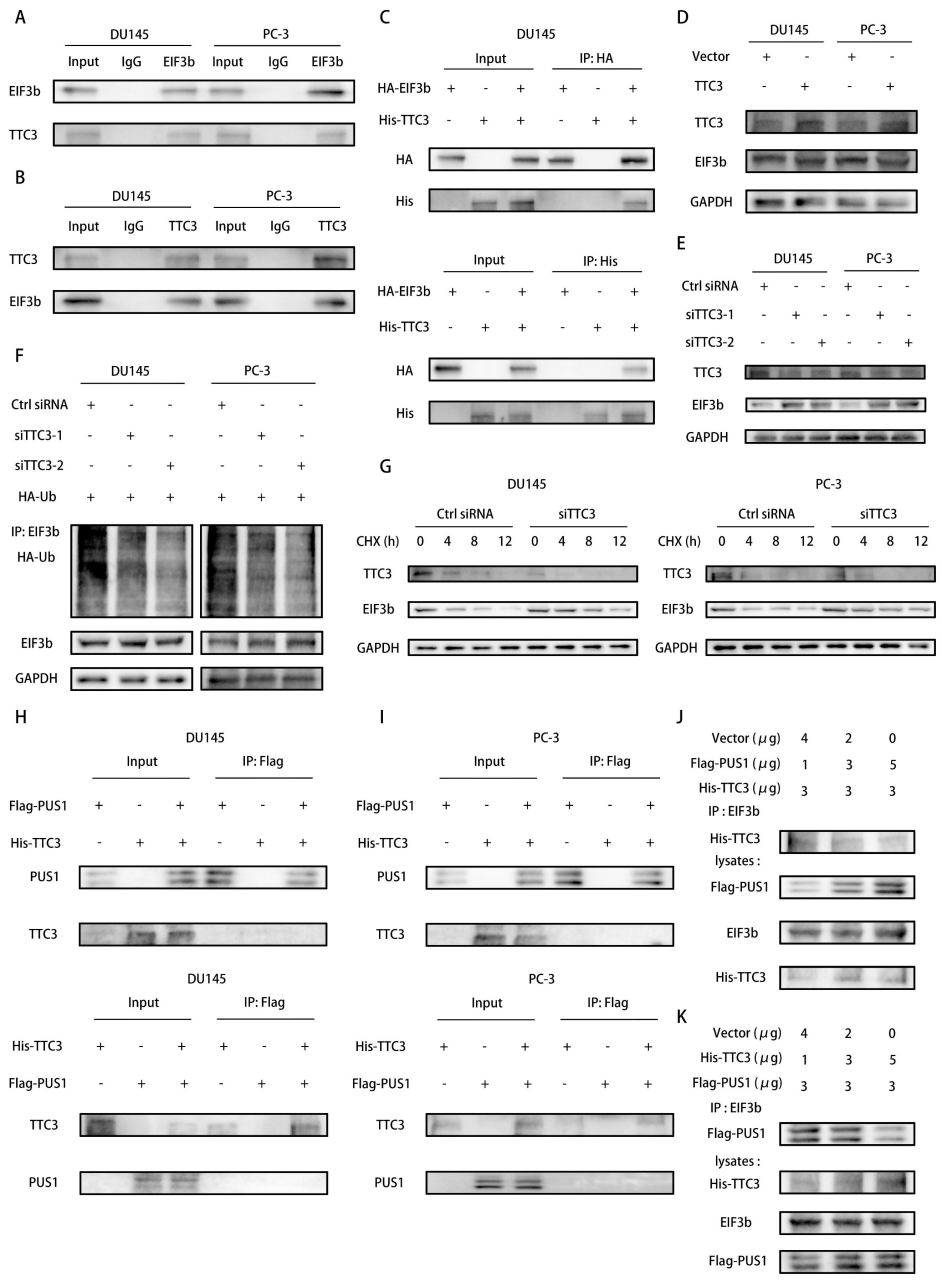
PUS1 Protects EIF3b from TTC3-Mediated Ubiquitination Degradation Through Competitive Binding. **A&B** Immunoprecipitation analysis showing the interaction between endogenous TTC3 and EIF3b in DU145 (left) and PC-3 (right) cells. **C** Immunoprecipitation analysis of the interaction between exogenous EIF3b and TTC3 in DU145 cells transfected with HA-EIF3b and His-TTC3. **D&E** Western blot analysis of EIF3b protein levels in DU145 and PC-3 cells after overexpression (D) or knockdown (E) of TTC3. **F** Immunoprecipitation analysis of ubiquitination levels of EIF3b in DU145 and PC-3 cells after knockdown of TTC3, and co-transfection with HA-Ub and EIF3b plasmids. **G** Western blot analysis of EIF3b protein levels at different time points in DU145 and PC-3 cells transfected with si-NC or si-TTC3 after treatment with cycloheximide (CHX, 20 μg/mL). **H&I** Immunoprecipitation analysis showing the interaction between endogenous PUS1 and TTC3 in DU145 (left) and PC-3 (right) cells. **J&K** HEK-293T cells were co-transfected with the specified plasmids and cultured for 48 hours, followed by Co-IP analysis using an anti-EIF3b antibody.

Indeed, E3 ubiquitin ligases facilitate the ubiquitination degradation of target proteins by linking them to the ubiquitin molecule^33^. To elucidate which E3 ubiquitin ligase functions in the ubiquitination degradation of EIF3b, we conducted EIF3b protein immunoprecipitation followed by mass spectrometry analysis, revealing the involvement of TTC3 in this process (Fig. S5A&B). Co-immunoprecipitation experiments further validated the interaction between TTC3 and EIF3b (Fig. 6A&B). Moreover, we confirmed the correlation between exogenous EIF3b and TTC3, and vice versa (Fig. 6C). To investigate whether EIF3b serves as a substrate for TTC3-mediated ubiquitination degradation, we transiently transfected TTC3 into tumor cells and observed a significant decrease in endogenous EIF3b protein levels (Fig. 6D). Conversely, transient knockdown of TTC3 using siRNAs resulted in a marked increase in EIF3b protein levels (Fig. 6E). Furthermore, we found that TTC3 knockdown significantly increased EIF3b protein stability, indicating TTC3's involvement in this process. Subsequently, we assessed the polyubiquitination levels of EIF3b after TTC3 knockdown. As expected, TTC3 knockdown markedly inhibited the ubiquitination levels of EIF3b (Fig. 6F&G and (Fig. S5C&D).

In summary, we discovered that both PUS1 and TTC3 can influence the ubiquitination levels of EIF3b. Therefore, we speculate that PUS1 may protect EIF3b from TTC3-mediated ubiquitination degradation by affecting the binding between TTC3 and EIF3b. To verify this hypothesis, we transfected Flag-PUS1 and His-TTC3 into PC-3 and DU145 cells, and co-immunoprecipitation revealed no interaction between them (Fig. 6H&I). Subsequently, we co-transfected Flag-PUS1, His-TTC3, and HA-EIF3b into 293T cells and found that overexpression of PUS1 significantly inhibited the interaction between EIF3b and TTC3, while overexpression of TTC3 also reduced the interaction between PUS1 and EIF3b (Fig. 6J&K). In conclusion, PUS1 competitively binds to EIF3b with TTC3, thereby protecting EIF3b from TTC3-mediated ubiquitination degradation.

### FOXA1 acts as an upstream transcription factor of PUS1 and facilitates its expression in prostate cancer cells

**Figure 7:**
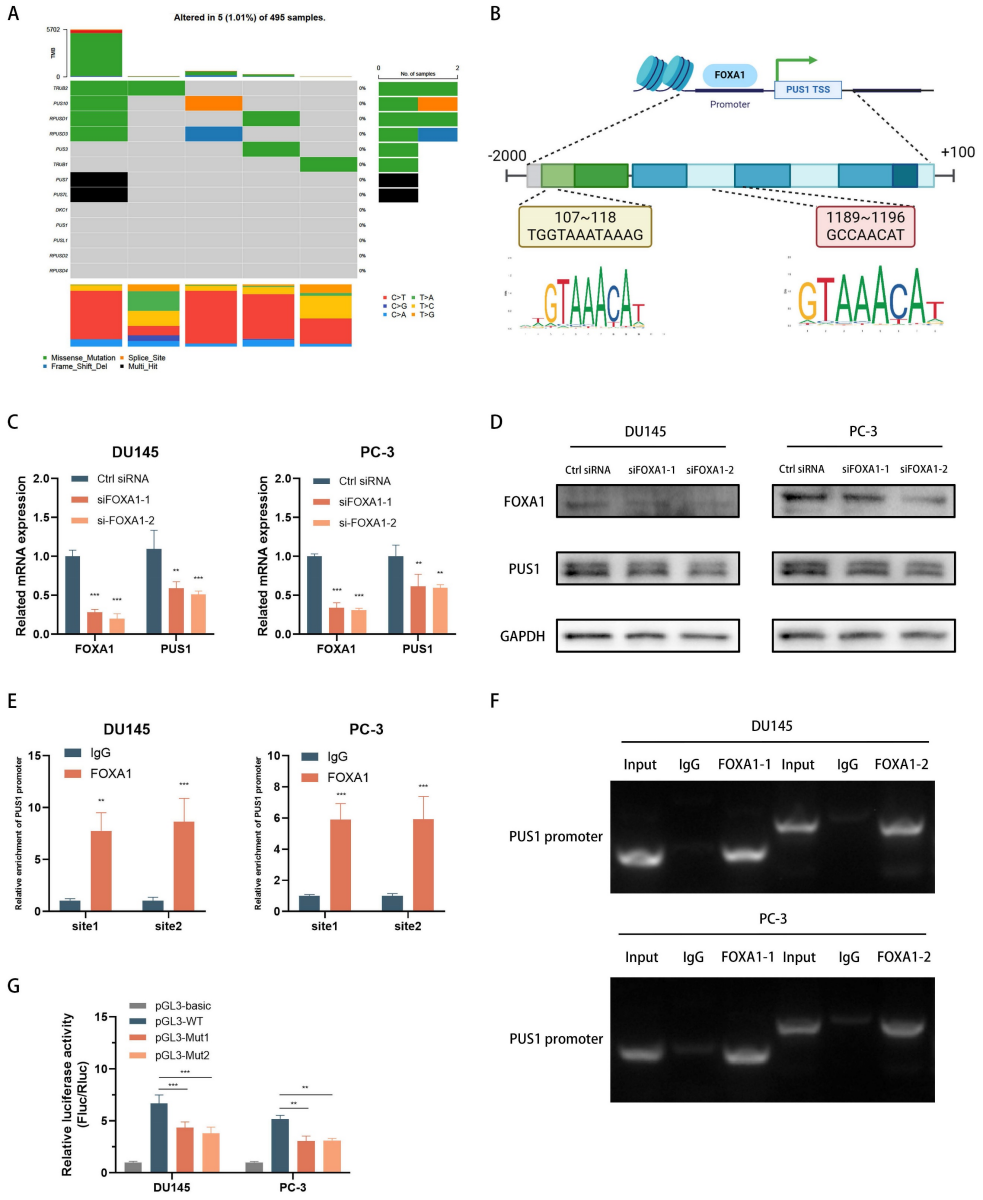
FOXA1 Acts as an Upstream Transcription Factor of PUS1 and Facilitates its Expression in Prostate Cancer Cells. **A** Mutation data for the pseudouridine modification enzymes in TCGA-PRAD. **B** Schematic diagram of the predicted binding sites for FOXA1 within 2000 bp upstream and 100 bp downstream of the PUS1 gene (promoter region). **C&D** FOXA1 and PUS1 expression in DU145 and PC-3 cells after FOXA1 knockdown using qRT-PCR (C) and western blot (D) analysis. **E&F** ChIP experiment to identify FOXA1 binding sites on the PUS1 promoter, followed by validation using qRT-PCR and DNA gel electrophoresis. **G** Bar graphs of relative luciferase activity in DU145 and PC-3 cells treated as indicated. **p<0.01, ***p<0.001.

To gain deeper insights into the importance of PUS1 in regulating EIF3b and promoting prostate cancer cell bone metastasis, we integrated data from the TCGA database on PUS1 mutations in prostate cancer. Our analysis revealed that PUS1 does not exhibit significant mutations in tumor samples (Fig. 7A). Consequently, we hypothesized that transcription factors might play a crucial role in this regulatory process. To explore this hypothesis further, we focused on identifying and characterizing transcription factors potentially involved in regulating PUS1 expression. Using NCBI to query the promoter region of PUS1, we selected a region extending 2kb upstream and 100bp downstream of the PUS1 gene (Fig. 7B). By combining data derived from the UCSC Genome Browser, JASPAR prediction tool, and KnockTF database, we initially identified six transcription factors: FOXA1, GABPA, GATA2, SRF, MAP3K7, and E2F2. Subsequent analysis revealed that only FOXA1, a potential regulator of PUS1, was significantly upregulated in prostate cancer tissues (Fig. S6A-F). We then aimed to validate whether FOXA1 acts as a transcriptional regulator of PUS1.

In DU145 and PC-3 cell lines, knockdown of FOXA1 led to a significant decrease in PUS1 expression, as demonstrated by qPCR and Western blot analyses (Fig. 7C&D). To further investigate how FOXA1 regulates PUS1, we performed ChIP and DNA gel electrophoresis experiments, which showed that FOXA1 binds to the PUS1 gene at upstream regions 107-118bp and 1189-1196bp (Fig. 7E&F). Additionally, dual-luciferase assays revealed that mutation of the FOXA1 binding sites in the PUS1 promoter region significantly reduced luciferase activity (Fig. 7G). These results collectively indicate that FOXA1 can bind to the promoter region of PUS1 and drive its transcription.

### Targeting PUS1 with mogroside IV-E suppresses *in vitro* invasion and migration of prostate cancer cells, and *in vivo* bone metastasis

**Figure 8:**
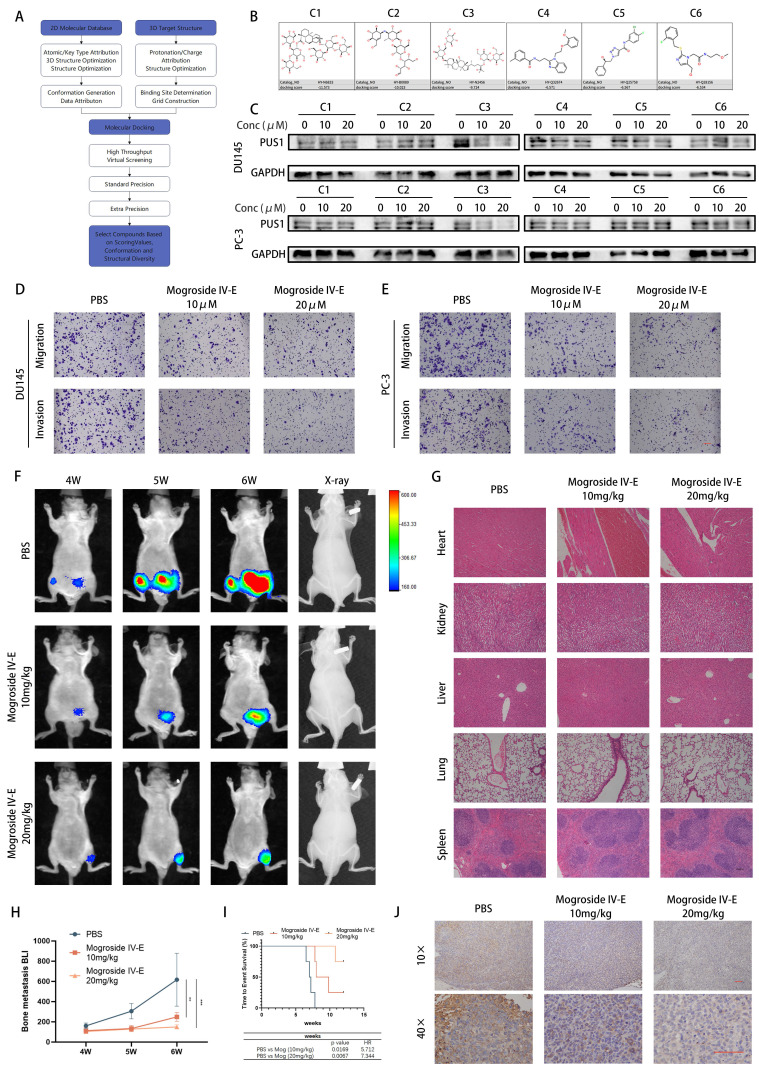
Targeting PUS1 with Mogroside IV-E Suppresses *In Vitro* Invasion and Migration of Prostate Cancer Cells, and *In Vivo* Bone Metastasis. **A** Schematic diagram of the screening process for PUS1 small molecule inhibitors. **B** Chemical structures of the top 3 small molecule inhibitors from each of the two compound libraries. **C** Quantitative assessment of PUS1 protein expression using Western blotting in DU145 and PC-3 cells after 48-hour treatment with PBS, 10 μM, and 20 μM concentrations of each of the 6 compounds. **D&E** Representative transwell invasion and migration assay images of DU145 and PC-3 cells treated as indicated. Scale bar, 20 μm. **F** PC-3 cells carrying luciferase were injected into the tail artery of nude mice. Upon detecting significant bioluminescent signals, tumor-bearing mice were randomly divided into 3 groups (4 mice per group) and treated with the indicated dosages, and representative bioluminescence imaging results for each group of nude mice are shown. **G** Representative HE staining images of the heart, kidney, liver, lung, and spleen from treatment groups with different concentrations of Mogroside IV-E. Scale bar, 20 μm. **H** Bioluminescence quantification of bone metastatic lesions in different groups of nude mice. **I** Survival Kaplan-Meier curves for each group of mice. **J** Immunohistochemical staining of PUS1 as labeled. Scale bar, 20 μm. **p<0.01, ***p<0.001.

Given the current lack of small molecule inhibitors targeting PUS1 in the literature, we conducted a virtual screening of the MCE compound library and validated PUS1 inhibition based on the top three compounds from both libraries using molecular docking (Fig. 8A and Fig. S7A-B). Through western blot analysis, we observed significant inhibition of PUS1 expression by compound 3, namely Mogroside IV-E, showing a dose-dependent trend (Fig. 8B&C). Further investigation via transwell invasion and migration assays revealed alterations in the motility of tumor cells treated with 10uM and 20uM Mogroside IV-E. We found that Mogroside IV-E significantly inhibited the motility of prostate cancer cells, with these effects being more pronounced at higher concentrations (Fig. 8D&E and Fig. S7C).

Subsequently, we established a mouse model of bone metastasis by injecting PC-3-luc cells via the tail artery. After 3 weeks of injection, mice exhibited evident bone metastasis (Fig. S7D), and were then randomly divided into three groups: treated with PBS, 10mg/kg, or 20mg/kg Mogroside IV-E. As depicted in the figures, X-ray imaging revealed a significant reduction in bone destruction in the Mogroside IV-E treatment group compared to the PBS group. Additionally, the number of bone metastatic foci, fluorescence intensity and overall poor prognosis markedly decreased in the Mogroside IV-E treatment groups, showing a dose-dependent trend (Fig. 8F, H&I). Immunohistochemical analysis indicated a significant decrease in PUS1 expression in the Mogroside IV-E treatment group (Fig. 8J). Moreover, no apparent drug toxicity was observed in the hearts, livers, lungs, or kidneys of mice in the treatment groups (Fig. 8G and Table S8). In summary, our data demonstrate that Mogroside IV-E inhibits PUS1 expression in a dose-dependent manner and suppresses both *in vitro* and *in vivo* metastasis of prostate cancer cells.

## Discussion

Bone metastasis is the most common metastatic site of prostate cancer (PCa) and a major cause of poor prognosis^34^. However, the underlying mechanisms remain unclear. Previous studies have suggested a potential association between elevated levels of pseudouridine (Ψ) and PCa metastasis^35^, indicating that pseudouridine synthases may play a critical role in prostate cancer progression. The enzymatic activity of pseudouridine synthases in mediating tumor cell malignancy varies significantly across different cancers. For instance, in glioblastoma, high expression of PUS7 promotes tumorigenesis by targeting tRNA modifications to regulate codon-specific translation of key GSC regulators^10^. In colorectal cancer, pseudouridine synthase PUS7 promotes metastasis through a non-enzymatic pathway by regulating LASP1^15^. In clear cell renal cell carcinoma, PUS10 inhibits RCC migration through the PUS10/miR-194-5p/NUDC/Cofilin1 pathway, and this effect is independent of its catalytic activity^36^. Similarly, the RNA methyltransferase METTL16 facilitates the formation of the translation initiation complex and enhances transcript translation by interacting with eukaryotic initiation factors 3a and -b and ribosomal RNA through its Mtase domain, independent of its methyltransferase activity, thereby promoting hepatocarcinogenesis^37^.

In this study, we screened 13 known pseudouridine synthases and identified PUS1, demonstrating that PUS1 knockdown significantly inhibited *in vivo* and *in vitro* metastasis without markedly altering global RNA pseudouridylation levels. To determine whether PUS1 influences tumor cell invasion and migration through its enzymatic activity, we established PUS1 wild-type and site-directed mutant stable cell lines (mutations including D146A, Y201F, I294L, R295K, and L333I)^32^. Both wild-type and mutant PUS1 significantly enhanced tumor cell migration compared to controls, and the RNA pseudouridylation levels in the mutant group were similar to controls, indicating that PUS1 primarily functions through a non-enzymatic pathway in prostate cancer cells. Further investigation using co-immunoprecipitation and immunofluorescence techniques revealed and confirmed that PUS1 interacts with EIF3b.

EIF3b, a subunit of the eukaryotic initiation factor 3 complex, has been implicated in metastasis in prostate cancer and other solid tumors. Previous research has focused primarily on downstream regulation of EIF3b, with limited understanding of its regulatory mechanisms in tumor progression.

Our study elucidates that EIF3b is a downstream target of PUS1 and mediates the pro-metastatic function of PUS1. PUS1 stabilizes EIF3b by reducing its ubiquitin-mediated degradation. E3 ubiquitin ligases play a crucial role in protein degradation^33^. We demonstrated the involvement of TTC3 in the ubiquitin-mediated degradation of EIF3b based on three pieces of evidence. Firstly, EIF3b interacts with TTC3. Secondly, TTC3 reduces the half-life of the EIF3b protein. Lastly, TTC3 increases the ubiquitination levels of EIF3b. These findings indicate that TTC3 acts as a negative regulator of EIF3b, contrary to the function of PUS1. Therefore, we explored whether PUS1 and TTC3 influence each other's affinity for EIF3b. Co-IP experiments showed no direct interaction between PUS1 and TTC3, but overexpression of PUS1 significantly inhibited TTC3 binding to EIF3b, and vice versa.

Notably, although TTC3 has been reported as an E3 ubiquitin ligase in other studies^38^-^40^, its role in the ubiquitin-mediated degradation of EIF3b has not been elucidated. Our study is the first to reveal that TTC3 not only participates in EIF3b ubiquitination but also plays a critical role in this process. Previous research has shown that TTC3, as an E3 ubiquitin ligase, plays a role in biological processes, including the regulation of the cell regulation^41^ and neural development^42^. However, our study is the first to detail the specific role of TTC3 in EIF3b ubiquitination, providing new insights into the regulatory mechanisms of EIF3b in tumor progression. Although PUS1 and TTC3 competitively bind to EIF3b, further research is needed to determine whether they interact with the same domains of EIF3b.

Despite the observed upregulation of PUS1 in metastatic prostate cancer, the mechanisms underlying its upregulation remain unclear. Integrative analysis of gene expression and mutation data from the TCGA database revealed no significant mutation rate of PUS1 in tumor samples, suggesting that its upregulation is not mutation-driven. Considering the complexity of gene expression regulation, it is plausible that transcription factors play a key role. In this project, we identified FOXA1 as a transcription factor for PUS1, promoting its transcriptional activation by binding to specific sequences in the promoter region. ChIP and DNA Gel Electrophoresis showed significant binding of FOXA1 to the upstream regions of the PUS1 promoter at positions 107-118 and 1189-1196 bp. Furthermore, dual-luciferase assays confirmed that FOXA1 significantly increased PUS1 promoter activity.

Compared to siRNA, small molecule inhibitors are more selective and cell-permeable, making them more feasible for *in vivo* gene targeting. Targeting the FOXA1/PUS1/EIF3b signaling axis, we screened the MCE compound library and identified Mogroside IV-E as a small molecule inhibitor of PUS1. Western blot analysis showed that Mogroside IV-E inhibited PUS1 expression in a concentration-dependent manner. *In vitro* assays confirmed that tumor cell invasion and migration decreased in a dose-dependent manner with Mogroside IV-E treatment. To verify its *in vivo* efficacy, we conducted a mouse tail artery injection model, finding that the results were consistent with the *in vitro* findings. Additionally, the drug-treated group showed no significant toxicity in the lungs, heart, kidneys, liver, or spleen compared to the control group. Collectively, these results suggest that targeting PUS1 with Mogroside IV-E may represent a promising therapeutic strategy for metastatic prostate cancer, as it not only inhibits tumor cell migration and invasion but also improves survival outcomes in xenograft mice.

While our study comprehensively elucidates that PUS1 interacts with EIF3b and protects EIF3b from TTC3-mediated ubiquitination, the molecular interactions between PUS1 and EIF3b require further investigation. Constructing truncated versions of PUS1 and EIF3b will help identify their interaction domains. Secondly, studying the downstream effects of EIF3b stabilization will provide deeper insights into the metastatic process by identifying specific mRNAs and proteins regulated by stabilized EIF3b. Thirdly, although we demonstrated the phenotypic effects of the small molecule inhibitor Mogroside IV-E on prostate cancer metastasis, its precise mechanisms and pharmacokinetics warrant further exploration.

## Conclusions

In summary, our study confirms that PUS1 interacts with EIF3b, suppressing TTC3-mediated ubiquitination degradation, thereby identifying PUS1 as a novel protective molecule of EIF3b, mediating prostate cancer metastasis both *in vitro* and *in vivo*. These data highlight a non-enzymatic role of PUS1 in tumor migration and invasion. Furthermore, targeting PUS1 with Mogroside IV-E offers a new therapeutic approach and means for the prevention and treatment of prostate cancer bone metastasis.

## Supplementary Material

Supplementary figures and tables.

## Abbreviations

PCa: Prostate cancer
FBS: Fetal bovine serum
PRAD: Prostate adenocarcinoma
BPH: Benign prostatic hyperplasia
PUS1: Pseudouridine Synthase 1
EIF3b: Eukaryotic Translation Initiation Factor 3 Subunit B
TTC3: Tetratricopeptide Repeat Domain 3
FOXA1: Forkhead Box A1
Baf: Bafilomycin
IF: Immunofluorescence
CHX: Cycloheximide
WB: Western blot assay
ChIP: Chromatin immunoprecipitation
IHC: Immunohistochemistry
IRS: Immunoreactivity score
OS: Overall survival
DFS: Disease-free survival
PFS: Progression-Free Survival
HR: Hazard ratios
CI: Confidence intervals
SYSMH: Sun Yat-sen Memorial Hospital
