# Supplementary material for: FOXA1-dependent PUS1 regulates EIF3b stability in a non-enzymatic pathway mediating prostate cancer bone metastasis
Source: Int J Biol Sci. 2024 Aug 19;20(11):4566–84. doi: 10.7150/ijbs.100905 (PMC11380452; doi:10.7150/ijbs.100905)
Supplement: Supplementary file 1 — Supplementary figures and tables. [file ijbsv20p4566s1.pdf]

Supplementary file

Table 1 PUS1 expression and clinicopathologic characteristics of prostate cancer patients in TCGA, Cohort 1 and Cohort 2.

Variables		TCGA	Cohort 1	Cohort 2
		Number of cases (%)	Number of cases (%)	Number of cases (%)
Age	≤60	228 (45.1%)	33 (22.0%)	2 (6.7%)
	> 60	277 (54.9%)	127 (78.0%)	28 (93.3%)
Gleason score	≤3+4	188 (43.2%)	63 (42.0%)	-
	≥4+3	247 (56.8%)	87 (58.0%)	-
T stage	T1+2	228 (46.6%)	77 (51.3%)	-
	T3+4	261 (53.4%)	73 (48.7%)	-
N status	N0	411 (83.7%)	128 (85.3%)	-
	N1	80 (16.3%)	22 (14.7%)	-
PUS1	Low	124 (25.4%)	111 (74.0%)	-
	High	365 (74.6%)	39 (26.0%)	-

Table 2 Antibodies used in this study.

Antibody name	Cat No.	Brand
PUS1	ab203010	Abcam
EIF3b	10319-1-AP	Proteintech
FOXA1	53528S	Cell Signaling Technology
TTC3	ab80061	Abcam
Rabbit IgG control	30000-0-AP	Proteintech
GAPDH	60004-1-Ig	Proteintech
Flag-tag	66008-4-Ig	Proteintech
HA-tag	3724S	Cell Signaling Technology
His-tag	12698	Cell Signaling Technology
Ubiquitin	20326S	Cell Signaling Technology

Table 3 Primers used in this study.

Primer	Forward	Reverse
PUS1	TCAACAGCCACCTTCCCTCTCA	GCAATAGGTCCTGGCATCACATC
EIF3b	ACAAGCAGCAGGCGAACACCAT	TCCACAAACGCTAAGGCACCGT
FOXA1	GCAATACTCGCCTTACGGCTCT	GGGTCTGGAATACACACCTTGG
GAPDH	GTCTCCTCTGACTTCAACAGCG	ACCACCCTGTTGCTGTAGCCAA
PUS1-promoter-ChIP-1	TCCATTCACCTGCCGACATA	AGTGTGTGCAGGGATGAGG

PUS1-promoter-ChIP-2	AGTGGGCCGGTCTCTTAAAA	AAGCGATTCTCCCGTCTCAG

Table 4 Patient characteristics for RNA-sequencing.

Patient	Age	TNM stage	Gleason score
Primary 1	69	T3aN0M0	3+4
Primary 2	69	T3bN0M0	4+4
Primary 3	68	T3bN0M0	4+4
Primary 4	73	T3aN0M0	4+3
Primary 5	80	T3aN0M0	4+4
Primary 6	68	T3aN0M0	4+5
Primary 7	57	T3bN0M0	4+3
Primary-BM 1	74	T4N1M1b	4+4
Primary-BM 2	73	T4N1M1b	4+5
Primary-BM 3	70	T4N0M1b	4+4
Primary-BM 4	74	T4N0M1b	4+5
Primary-BM 5	68	T4N1M1b	4+4
Primary-BM 6	65	T4N0M1b	4+5

Table 5 Sequences of siRNA oligos and shRNAs used in this study.

	Sense	Antisense
Negative Control	UUCUCCGAACGUGUCACGUTT	ACGUGACACGUUCGGAGAATT
PUS1-siRNA1	GCUUCAUGAUGCAUCAGAUTT	AUCUGAUGCAUCAUGAAGCUC
PUS1-siRNA2	UCGGGUCCUCACAAUUCAATT	UUGAAUUGUGAGGACCCGACA
EIF3b-siRNA1	GCCUCCUGCAGAAGAACAATT	UUGUUCUUCUGCAGGAGGCTT
EIF3b-siRNA2	GCACCUACCUGGCUACCUUTT	AAGGUAGCCAGGUAGGUGCTT
FOXA1-siRNA1	GCACUGCAAUACUCGCCUUTT	AAGGCGAGUAUUGCAGUGCTT
FOXA1-siRNA2	GGACUUCAAGGCAUACGAATT	UUCGUAUGCCUUGAAGUCCTT
TTC3-siRNA1	GGAGCACCAAGUAUUACAAGACCAATT	UUGGUCUUGUAAUACUUGGUGCUCCTT
TTC3-	GCAGUAUGCUGACAAGAUUAAA	GGAUUUAAUCUUGUCAGC

siRNA2	UCCTT	AUACUGCTT
PUS1-shCtrl	CCTAAGGTTAAGTCGCCCTCG	
PUS1-shRNA1	CCGGGCTTCATGATGCATCAGAT CTCGAGATCTGATGCATCATGAA GCTTTTTGAATT	
PUS1-shRNA2	CCGGTCGGGTCCTCACAATTCAA CTCGAGTTGAATTGTGAGGACCC GATTTTTGAATT	

Table 6 Binding Sequences of FOXA1 and PUS1 Promoter Regions and Their Mutant Variants.

	**Sequence**
Site 1	TGGTAAATAAAG
Site 2	GCCAACAT
Site 1 mutation	ACCATTTATTTC
Site 2 mutation	CGGTTGTA

Table 7 Univariate and multivariate Cox regression analyses of disease-free survival in TCGA.

Variables	Univariate		Multivariate	
	HR (95% CI)	P value	HR (95% CI)	P value
PUS7L	1.022(0.720−1.450)	0.903	1.240(0.548−2.807)	0.605
PUS7	1.249(0.909−1.717)	0.171	1.439(0.767−2.700)	0.256
PUS3	1.237(0.728−2.103)	0.432	0.898(0.406−1.986)	0.791
PUS10	0.888(0.579−1.360)	0.584	0.716(0.330−1.556)	0.399
RPUSD2	1.444(0.818−2.548)	0.205	0.804(0.385−1.680)	0.562
TRUB1	0.998(0.771−1.290)	0.986	1.458(0.734−2.897)	0.282
PUS1	2.068(1.386−3.087)	<0.001	2.361(1.151−4.843)	0.019
RPUSD3	2.057(0.989−4.277)	0.053	0.990(0.395−2.480)	0.983
PUSL1	1.688(1.158−2.460)	0.006	1.298(0.725−2.323)	0.380
RPUSD1	1.778(1.203−2.627)	0.004	1.047(0.518−2.116)	0.897
RPUSD4	1.413(0.857−2.328)	0.175	0.847(0.393−1.827)	0.672
DKC1	1.249(0.812−1.922)	0.311	0.309(0.102−0.937)	0.038
TRUB2	1.988(1.158−3.413)	0.013	2.266(0.918−5.592)	0.076

Table 8 Effects of Mogroside IV-E on blood in vivo.

**Blood Index**	**PBS (Mean±SD)**	**Mogroside IV-E 10mg/kg**	**Mogroside IV-E 20mg/kg**	**Reference Value**

		**(Mean±SD)**	**(Mean±SD)**	
HGB	138.75±7.63	122.50±8.81	119.75±5.25	110-165 g/L
WBC	8.55±1.90	4.83±1.65	6.48±1.40	0.8-10.6×10^9/L
Granulocyte%	14.43±9.05	18.43±6.07	26.35±9.63	6.5-50 %
ALT	31.74±5.41	42.51±7.40	41.28±5.81	10.06-96.47 U/L
AST	106.33±19.36	140.89±10.37	160.36±10.70	36.31-235.48 U/L
CREA	21.73±4.03	23.30±2.91	21.57±3.21	10.91-85.09 umol/L
ALP	108.94±8.86	115.99±5.26	97.23±27.36	22.52-474.35 U/L
BUN	29.48±1.28	29.05±3.59	29.27±2.13	10.81-34.74 mg/dL

Figures and legends

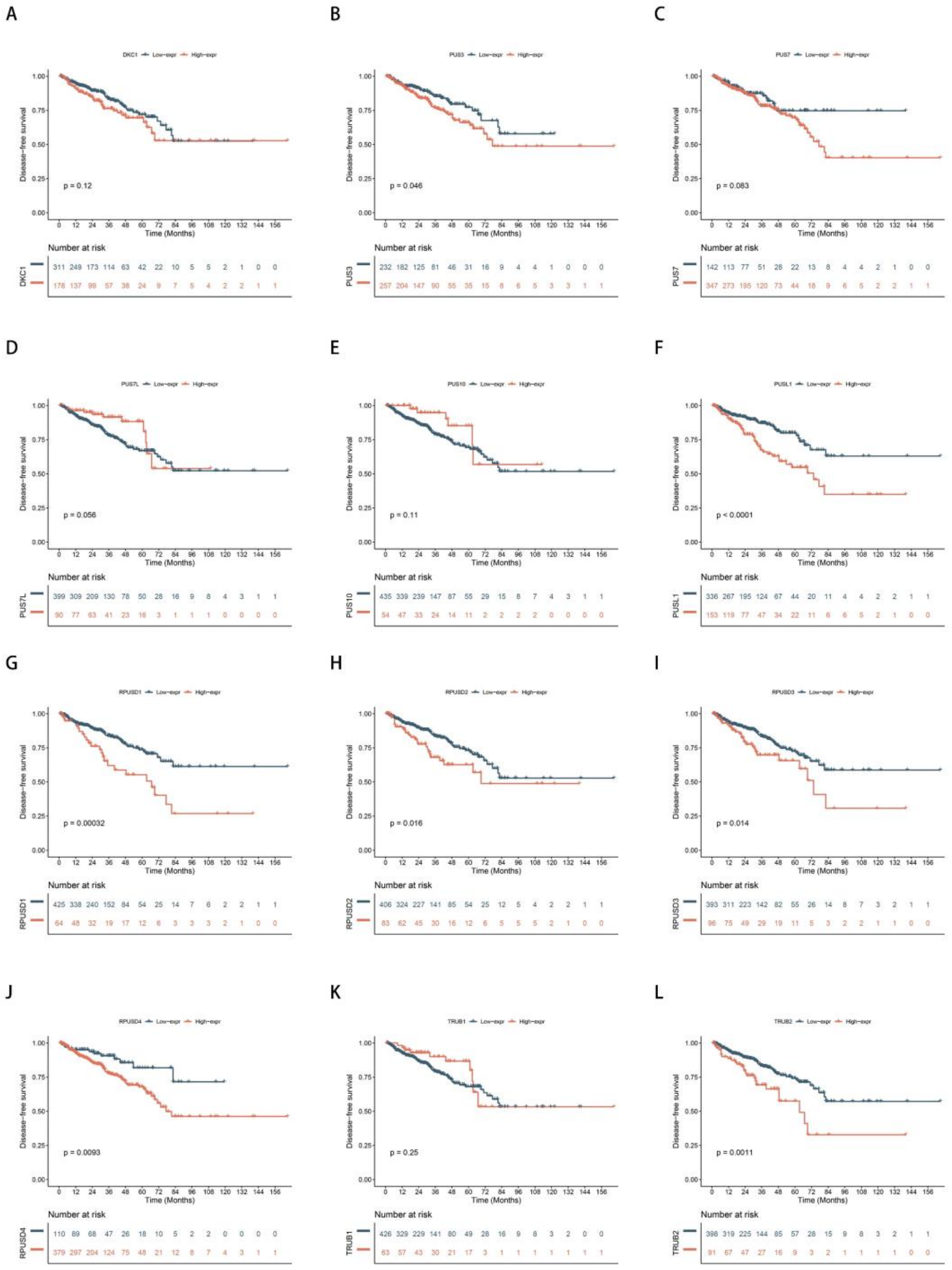

Fig. S1 DFS of the 12 pseudouridine synthases in TCGA-PRAD. **A-L** DFS of patients with different DKC1, PUS3, PUS7, PUS7L, PUS10, PUSL1, RPUSD1-4, TRUB1-2 expression levels.

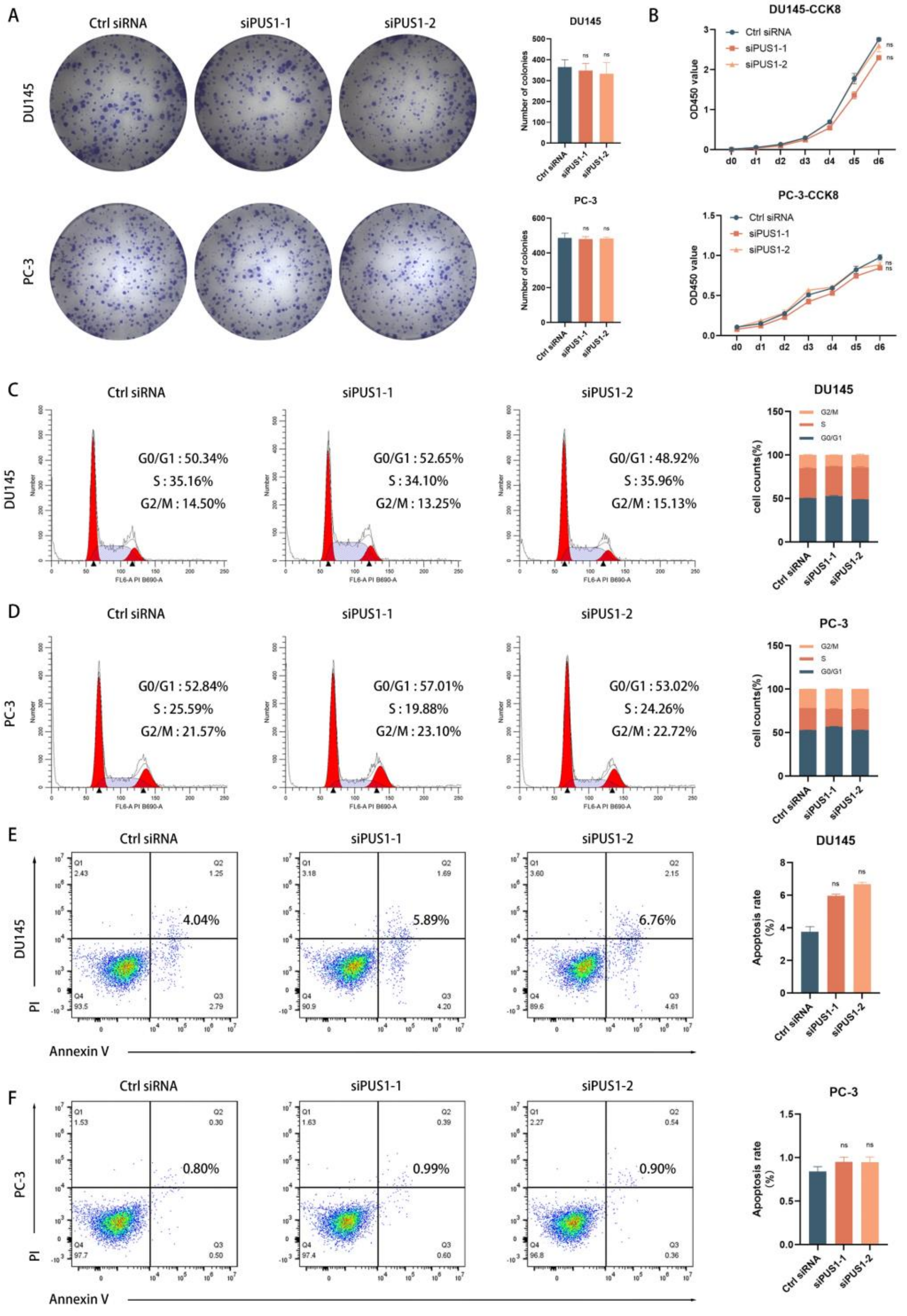

Fig. S2 Knockdown of PUS1 does not affect tumor cell proliferation, cell cycle, or apoptosis. **A&B** Representative images and bar graphs of colony formation assays (A) and CCK-8 proliferation assays (B) in DU145 and PC-3 cells treated as indicated. **C&D** Representative images and bar graphs of flow cytometry cell cycle assays in DU145 (C) and PC-3 (D) cells treated as indicated. **E&F** Representative images and bar graphs of flow cytometry cell apoptosis assays in DU145 (E) and PC-3 (F) cells treated as indicated. Data are presented as the mean ± SD of three independent experiments. ns: no significance.

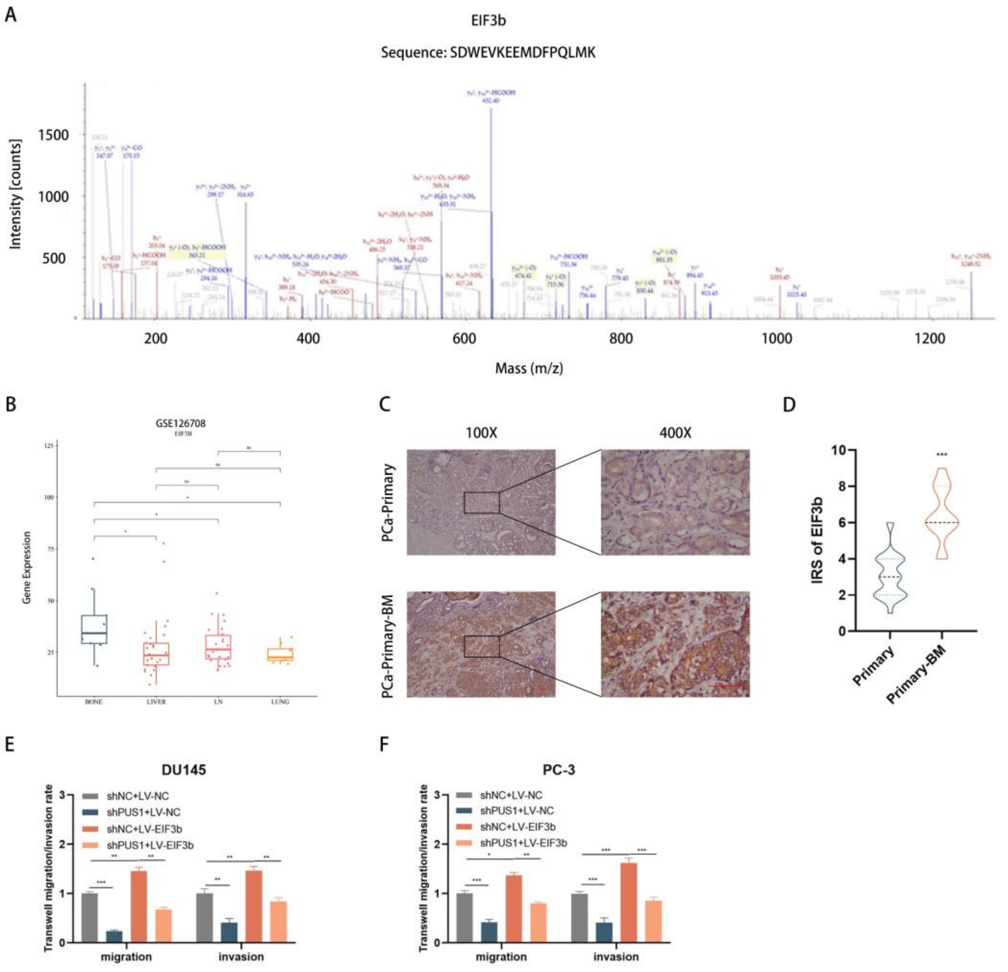

Fig. S3 PUS1 knockdown could be rescued by overexpressing EIF3b. **A** Mass spectrometry-identified representative peptide of EIF3b. **B** EIF3b expression levels in different metastatic sites of prostate cancer (GSE12708). **C&D** Representative immunohistochemical staining images of EIF3b and corresponding IRS score statistics in primary prostate cancer without metastasis, and primary prostate cancer with bone metastasis. Scale bar, 20 μm. **E&F** Bar graphs of transwell migration/invasion assays in DU145 (A) and PC-3 (B) cells treated as indicated. ns: no significance. * $p < 0.05$, ** $p < 0.01$, *** $p < 0.001$.

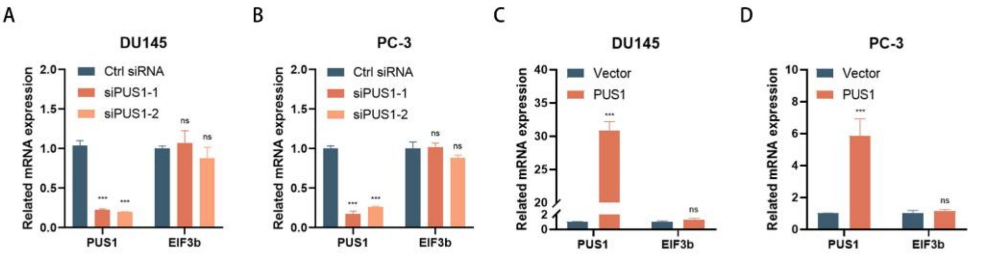

Fig. S4 EIF3b mRNA levels are not altered following PUS1 knockdown or overexpression. **A-D** Bar graphs of relative PUS1 and EIF3b mRNA levels in DU145 (A&C) and PC-3 (B&D) cells treated as indicated. ns: no significance. *** $p < 0.001$.

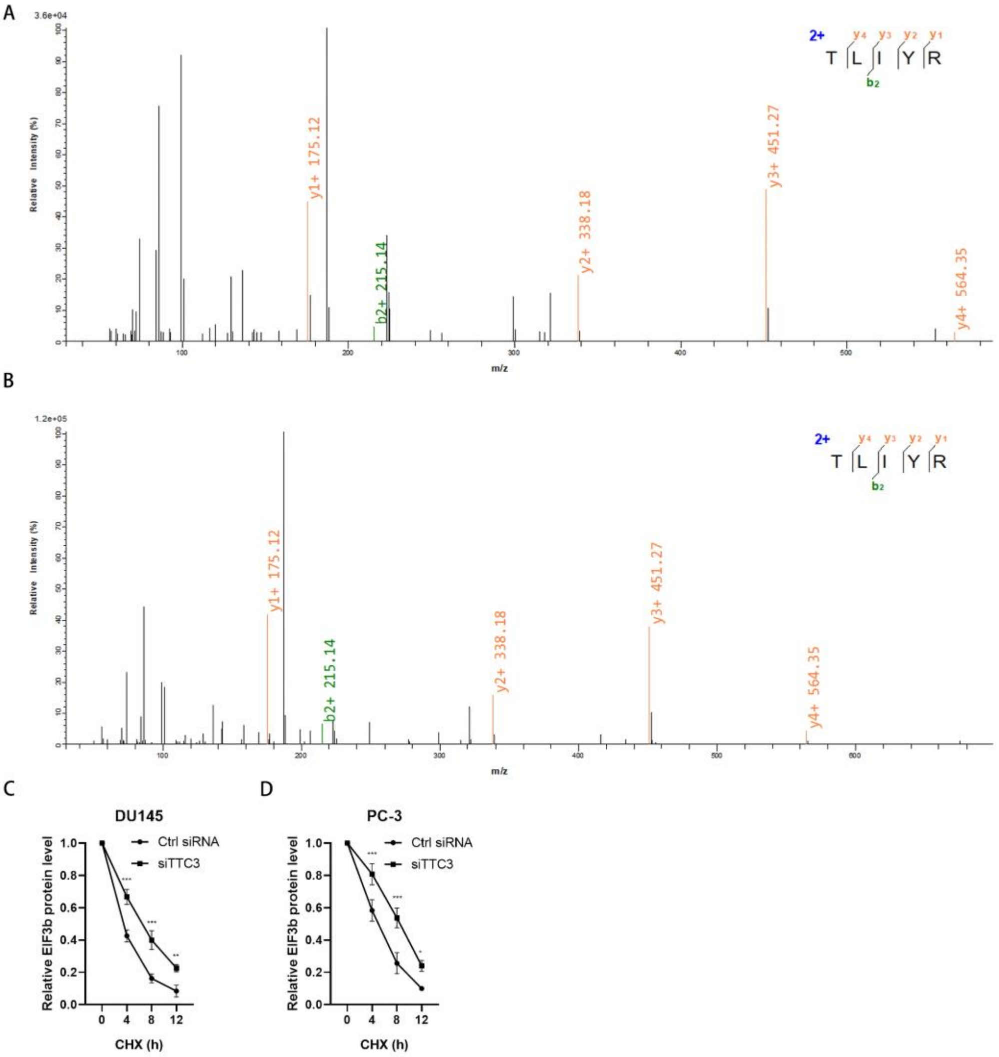

Fig. S5 EIF3b interacts with TTC3. **A&B** Mass spectrometry-identified representative peptide of TTC3. **C&D** Statistical analysis of EIF3b protein expression as indicated. * $p < 0.05$, ** $p < 0.01$, ***$p < 0.001$.

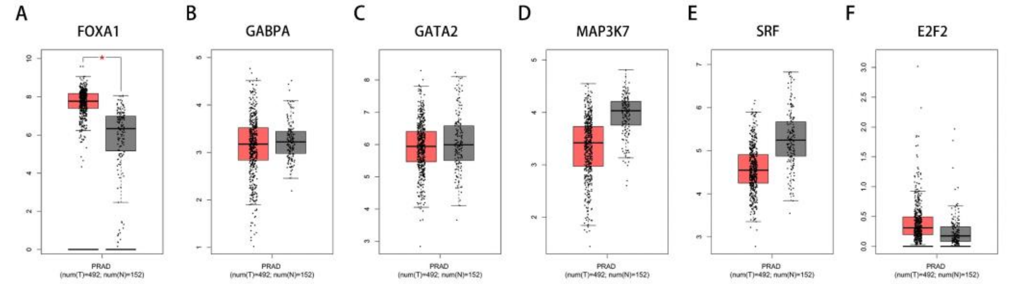

Fig. S6 Transcription factors of PUS1 predicted by database and their expression in prostate cancer. **A-F** Bar Graphs Showing Tumor versus Normal Tissue Expression in TCGA-PRAD for FOXA1, GABPA, GATA2, SRF, MAP3K7, and E2F2. * $p < 0.05$.

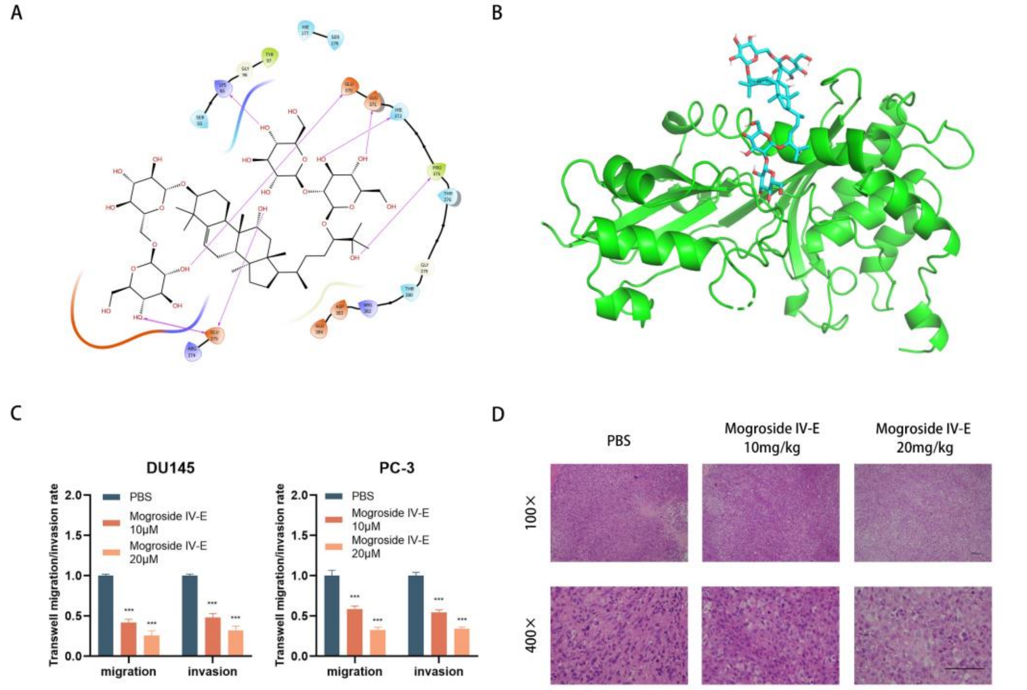

Fig. S7 Mogroside IV-E inhibits prostate cancer cell metastasis in vivo and in vitro in a Dose-Dependent Manner. **A&B** 2D (A) and 3D (B) binding models of PUS1 with Mogroside IV-E. **C** Bar graph analysis of transwell migration/invasion assays in DU145 and PC-3 cells following the indicated treatments. **D** Representative HE staining of lesions in the indicated groups. Scale bar, 20 μm. ***$p < 0.001$.
